# *C*-Mannosylation Enhances the Structural Stability of Human RNase 2

**DOI:** 10.1016/j.isci.2020.101371

**Published:** 2020-07-16

**Authors:** Martin Frank, Daniela Beccati, Bas R. Leeflang, Johannes F.G. Vliegenthart

**Affiliations:** 1 Bijvoet Center, Division of Bio-Organic Chemistry, Utrecht University, Padualaan 8, Utrecht 3584 CH, The Netherlands; 2 Biognos AB, Box 8963, Göteborg 40274, Sweden

**Keywords:** Biochemistry, Structural Biology, Protein Structure Aspects

## Abstract

*C*-Mannosylation is a relatively rare form of protein glycosylation involving the attachment of an α-mannopyranosyl residue to C-2 of the indole moiety of the amino acid tryptophan. This type of linkage was initially discovered in RNase 2 from human urine but later confirmed to be present in many other important proteins. Based on NMR experiments and extensive molecular dynamics simulations on the hundred microsecond timescale we demonstrate that, for isolated glycopeptides and denatured RNase 2, the C-linked mannopyranosyl residue exists as an ensemble of conformations, among which ^1^C_4_ is the most abundant. However, for native RNase 2, molecular dynamics and NMR studies revealed that the mannopyranosyl residue favors a specific conformation, which optimally stabilizes the protein fold through a network of hydrogen bonds and which leads to a significant reduction of the protein dynamics on the microsecond timescale. Our findings contribute to the understanding of the biological role of *C*-mannosylation.

## Highlights

• NMR and MD show that *C*-linked mannose exists as an ensemble of conformations
• Conformation of mannose is influenced by the protein environment and solvent
• In RNase 2 mannose favors a conformation that optimally stabilizes the protein fold
• Efficient methods for analysis of a large number of MD trajectories are presented

## Graphical Abstract

**Figure:**
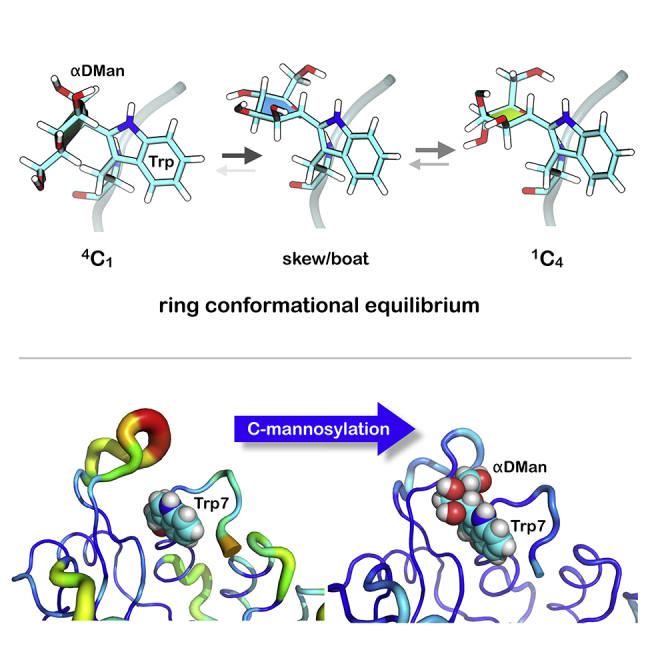

## Introduction

Glycosylation is one of the most common posttranslational modification of proteins, and the best known types are N- and O-glycosylation (Spiro, 2002). In the 1990s, C-glycosylation was discovered in human ribonuclease (RNase) 2, where an α-D-mannopyranosyl residue was attached to the C2 atom of the indole ring of Trp7 via a carbon-carbon bond (Hofsteenge et al., 1994; De Beer et al., 1995). In 1998, Krieg et al. proposed on the basis of site-directed mutagenesis the sequence Trp-X-X-Trp as the recognition motif for *C*-mannosylation, in which the Trp residue positioned toward the N terminus becomes mannosylated. It was later demonstrated that in thrombospondin type 1 repeats the second Trp residue is not essential for *C-*mannosylation (Gonzalez de Peredo et al., 2002) and can be replaced by Cys (Julenius, 2007).

*C*-mannosylation has been experimentally verified in approximately seventy glycosylation sites (Ihara et al., 2015) of proteins such as interleukin-12 (Yoon et al., 2000), the terminal four components of human complement system C6, C7, C8, and C9 (Hofsteenge et al., 1999), human platelet thrombospondin-1 (Hofsteenge et al., 2001), properdin (Hartmann and Hofsteenge, 2000), recombinant erythropoietin receptors (Furmanek et al., 2003), mucins MUC5AC and MUC5B (Perez-Vilar et al., 2004), MAG (Pronker et al., 2016), and hyaluronidase 1 (Goto et al., 2014). Screening of the human genome yielded about 2,600 exported or trans-membrane transcripts with at least one predicted *C*-Man site (Julenius, 2007), indicating that *C-*mannosylation is a more important protein modification than initially thought. It is now acknowledged that *C*-mannosylation is not limited to mammalian proteins but is present in the glycoprotein of the Ebola virus (Falzarano et al., 2007) and in the hypertrehalosemic hormone from the stick insect *Carausius morosus* (Munte et al., 2008).

*C*-mannosylation is a posttranslational modification that occurs intracellularly in the endoplasmic reticulum before protein folding and secretion (Krieg et al. 1997, 1998; Doucey et al., 1998) by a microsomal transferase previously isolated and characterized (Hamann et al., 1989). Its biological significance has not been unambiguously elucidated yet. It is believed that, when the polar mannose attaches to the non-polar tryptophan it affects the protein polarity, probably inducing a conformational change that affects protein functions such as protein stability, secretion, intracellular localization, and enzymatic activity (Goto et al., 2014).

Although direct involvement of *C*-mannose in adhesion phenomena of the W-X-X-W motif of thrombospondin type 1 repeat (TSR) modules is still being debated, Ihara et al. (2010) demonstrated, with the use of synthesized *C*-Man-WSPW and WSPW peptides, that in RAW264.7 cells some proteins specifically recognized *C*-Man-WSPW but not WSPW. Among these, the shock cognate protein 70 (Hsc70) had higher affinity in solution to *C*-mannosylated peptides compared with non-mannosylated ones. A study by Li et al. (2009) shows that *C*-mannosylated mindin from HEK 293 cells binds LPS through its TSR domain, whereas the bacterially expressed mindin that lacks *C*-mannose does not recognize LPS. Ihara et al. (2005) reported increased expression of *C*-mannosylation in the aortic vessels of diabetic Zucker rats, suggesting a pathological role for the increased *C-*mannosylation in the development of diabetic complications. Several studies demonstrated the biological importance of *C*-mannosylated tryptophan, although they could not investigate the contribution played by the *C*-mannose without modifying the target protein sequence. Otani et al. (2018) demonstrated that *C-*mannosylated tryptophan in G-CSFR plays a role in myeloid cell differentiation through regulation of downstream signaling. Sasazawa et al. (2015) showed how *C-*mannosylated tryptophan residues of the thrombopoietin receptor (c-Mpl) regulates thrombopoietin-dependent JAK-STAT signaling. Okamoto et al., 2017 revealed that mutagenesis of the *C*-mannosylation site reduced both secretion efficiency and enzymatic activity of *C-*mannosylation-defective mutant lipoprotein lipase-overexpressing cell lines compared with the wild-type. The identification of the gene encoding *C*-mannosyltransferase (Buettner et al., 2013), the enzyme affecting *C*-mannosylation, enabled genetic interventions aimed at modulating *C*-mannosylation without affecting target protein sequences and allowed studies that provided direct evidence for the specific role of *C*-mannosylation in the secretion of proteins containing TSR motif (Niwa et al., 2016; Shcherbakova et al., 2017).

The impact of *C*-mannosylation on the three-dimensional structure of proteins is not very well understood. Several studies have suggested changes in protein conformations due to the presence of *C-*mannose. Using NMR, Hinou et al., 2016 analyzed peptides synthesized to mimic the WSXWS motif of the erythropoietin receptor (EPOR) and compared them with the corresponding synthetic peptides lacking the *C*-mannose residue. Through analysis of NOEs signals, they were able to show that *C*-mannosylation works as a stabilizer of the WSXWS motif and the neighbor aryl side chain. Hartmann and Hofsteenge (2000) hypothesize that the 14 mannose residues linked to properdin are exposed at the surface of the protein in virtue of their hydrophilic character and mediate properdin interaction with the multivalent mannose-binding lectin present in serum. Tan et al. (2002) showed that the occurrence of several (*C*^*2*^-α-D-Man-)Trp residues in TSR modules poses steric constraints on the protein conformation. The polypeptide chain cannot adopt a helical conformation, and the mannosylated Trp residues are oriented so that their polar atoms are exposed and available for potential ligand binding. For MUC5AC and MUC5B (Perez-Vilar et al., 2004), ADAMTS-like 1 (Wang et al., 2009), and UNC5A (Shcherbakova et al., 2017) *C-*mannosylation is required for proper folding or subsequent endoplasmic reticulum exit. In TSR modules the *C*-mannosylated tryptophans are part of a 3D structural motif called “Trp-Arg ladder,” where up to three tryptophans (building a WxxWxxW motif) from one strand are stacked with conserved arginine residues (RxRxR motif) from a second strand. Such an arrangement of amino acid side chains is most likely stabilized by cation-π interactions. For properdin it has been shown by X-ray crystallography that the mannose can form hydrogen bonds with an adjacent arginine, which further stabilizes the Trp-Arg ladder (Pedersen et al., 2019). Such a stabilization has been recently confirmed experimentally for thrombospondin type 1 repeats of netrin receptor UNC-5 (Shcherbakova et al., 2019). However, it should be noted that a Trp-Arg ladder motif is not present in RNase 2.

**Figure 1:**
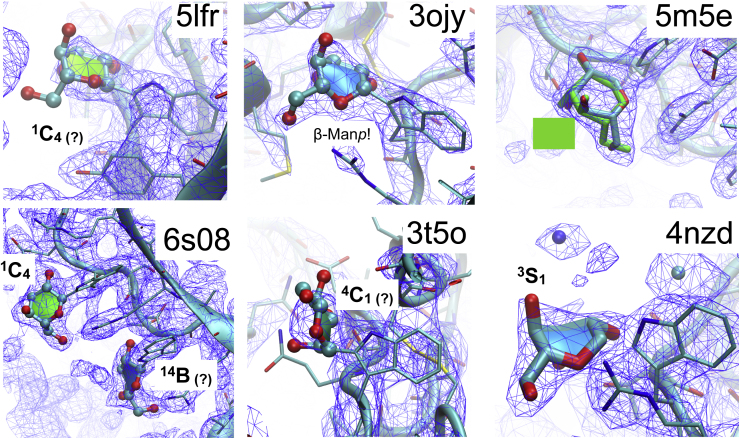
Examples of X-ray Crystal Structures Containing the α-D-Man*p*-Trp Moiety Well-resolved electron density supporting a ^1^C_4_ conformation of mannose is present in PDB entry 6s08 and 5m5e. Please note that in general the modeled ring shapes of carbohydrates in PDB structures should be taken with care (Agirre et al., 2017). As an example, PDB entry 5m5e is shown with a re-modeled mannose (shown in green) in ^1^C_4_ conformation, which fits at least equally well into the electron density than the distorted ring shape modeled in the original X-ray structure published. In most X-ray structures listed in Table S1 the electron density around the C-linked mannoses is not well resolved or the conformation of the mannose is ambiguous (indicated with a “?”). PDB entry 3ojy has β-D-Man*p* covalently attached to Trp, which is most likely not correct. The electron density in PDB entry 4nzd may support the existence of twisted ring shapes (skew, boat) of C-linked mannose. See also Table S1.

Following the initial discovery of *C-*mannosylation in RNase 2, extensive studies were conducted to identify the conformation of the *C*^2^-α-D-mannopyranosyl-L-tryptophan moiety (De Beer et al., 1995; Löffler et al., 1996). Since then, the X-ray structures of a number of *C-*mannosylated proteins have been determined; for example, interleukin-21 receptor bound to IL-21 (Hamming et al., 2012), interleukin-2 receptor (Klein et al., 2017), human complement system C6 (Aleshin et al., 2012), C5b6 (Hadders et al., 2012), C8 (Lovelace et al., 2011), MAG (Pronker et al., 2016), and properdin (van den Bos et al., 2019) (for a full list of available X-ray structures see Table S1). However, the electron density of the C*-*linked mannoses is frequently not sufficient to unambiguously determine their conformation (compare structures in Figure 1). Consequently, despite the availability of several X-ray structures, many structural aspects of the conformation of the α-D-Man*p*-Trp moiety remain unclear.

NMR spectroscopy revealed that the pyranose ring is not rigid in the hexapeptide FTW^Man^AQW isolated from RNase 2 (De Beer et al., 1995; Löffler et al., 1996) and adopts several conformations on the NMR timescale. Although there are some indications that the ^1^C_4_ conformation is the most prominent conformation in the ensemble, the other conformations present are unknown. Additionally, it has been shown that in human RNase 2 the orientation around the C-linkage occupied by the mannose residue in the native protein is different from that in the denatured form. The objective of the present study was to explore the conformation of the C*-*linked α-mannose by molecular modeling and to determine how the usual pyranose ^4^C_1_ conformation of the mannose residue may be destabilized in favor of the ^1^C_4_ conformation. To gain insight into the possible influence of the polypeptide chain on the conformation of the C*-*linked α-D-mannopyranosyl moiety, further NMR studies were carried out on glycopeptides of different chain length and amino acid composition.

NMR, molecular modeling, and molecular dynamics (MD) studies were performed on ManTrp, *C-*Man containing peptides, and both native and denatured RNase 2. Importantly, we investigated in which way the three-dimensional structure of RNase 2 could be responsible for the different orientation of *C*-mannose in native and denatured protein. Since RNase has the same amino acid sequence as eosinophil-derived neurotoxin (EDN) (Hamann et al., 1989), the three-dimensional structure of recombinant EDN (rEDN from *Escherichia coli*), as determined by X-ray crystallography (Mosimann et al., 1996; Swaminathan et al., 2002), was used to model the C-linkage between mannose and Trp. The short distances between the neighboring amino acid protons and those of the α-D-mannopyranosyl residue, as observed by molecular modeling, were finally compared with the NMR data on native RNase 2. Our results show that in native RNase 2 the mannopyranosyl residue favors a specific conformation, which optimally stabilizes the protein fold through a network of hydrogen bonds and which leads to a significant reduction of the protein dynamics.

## Results

### Structural Analysis of *C*^2^-α-D-Mannopyranosyl-L-Tryptophan by NMR in Peptides Isolated from Human RNase 2

**Figure 2:**
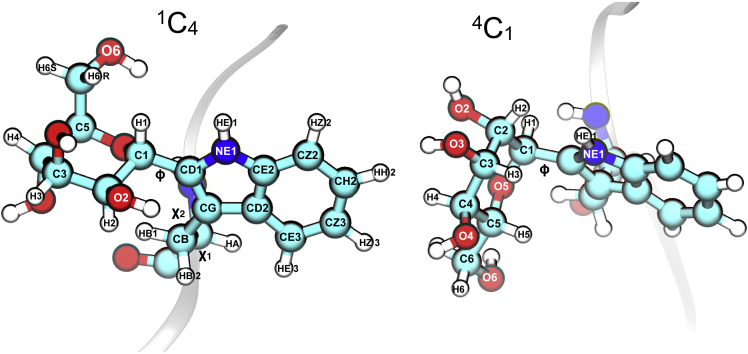
*C*^2^-α-D-mannopyranosyl-L-tryptophan Moiety 3D representations of two chair conformations: ^1^C_4_ (left), ^4^C_1_ (right). The peptide backbone is indicated as ribbon. Selected atom and torsion labels are shown. For definitions of torsion angles see Supplemental Information, Molecular Modeling section.

The structure and atom designation of *C*^2^-α-D-mannopyranosyl-L-tryptophan is presented in Figure 2. The complete NMR assignment of this moiety was first obtained from analysis of the *C-*glycopeptide FTW^Man^AQW isolated from human RNase 2 (Hofsteenge et al., 1994) and was later on confirmed by analysis of the (*C*^*2*^-α-D-Man-)Trp compound generated by total synthesis (Manabe and Ito, 1999). Previous studies indicate that the *C*-α-mannopyranosyl residue must exist in an ensemble of conformations on the NMR timescale (De Beer et al., 1995), among which the most represented is the ^1^C_4_ chair conformation with an equatorially oriented tryptophan moiety (Manabe and Ito, 1999).

To determine whether the (*C*^*2*^-α-D-Man-)Trp conformation could be influenced by the presence of neighboring amino acids, or whether the presence of the C-linked mannose could reduce the flexibility of the amino acid region linked to it, peptides with different chain length were here isolated from human RNase 2 and characterized by ^1^H NMR (Table 1). Chemical shifts and coupling constants of the (*C*^*2*^-α-D-Man-)Trp in the RNase 2 peptides were also compared with values for the chymotryptic peptide SSSW^Man^SEW isolated from human interleukin-12 (Doucey et al., 1999) and the tryptic peptide MSPW^Man^SEWSQCDPCLR isolated from the terminal component C9 of the human complement system (Hofsteenge et al., 1999). Although the reported fragments present different amino acid sequences, they closely resemble each other with respect to chemical shifts and coupling constants of the (*C*^*2*^-α-D-Man-)Trp moiety, indicating a similar conformation of the mannose ring. In all the reported peptides, Man ^3^*J*_1,2_ couplings constants present values around 7–8 Hz that are incompatible with the usually preferred ^4^C_1_ conformation of aldohexopyranoses and preclude all conformations with a small H1/H2 coupling constant (^4^C_1_, ^0,3^B, B_0,3_, B_1,4_, ^2,5^B, ^3^S_1_, ^5^S_1_, ^2^S_0_, see Table S2).

**Table 1:** ^1^H Chemical Shifts δ_H_ (ppm) and Homonuclear Vicinal ^1^H–^1^H Coupling Constants J_i,i+1_ (Hz) of the *C*-Glycosylated Amino Acid Residues in Isolated Peptides, Measured at 300 K

	W_Man_^a^		W^Man^AQW^a^		FTW^Man^AQW^a^^,^^b^		SSSW^Man^SEW^c^		MSPW^Man^SEW^d^
	δ_H_	J_i,i+1_	δ_H_	J_i,i+1_	δ_H_	J_i,i+1_	δ_H_	J_i,i+1_	δ_H_
Man H1	5.17	8.2	5.14	8.2	5.22	7.8	5.18	7.5	5.18
Man H2	4.42	3.2	4.40	3.1	4.42	3.2	4.44	3.0	4.44
Man H3	4.11	5.3	4.08	5.4	4.09	5.5	4.07	9.1^e^	4.07
Man H4	3.95	3.4	3.94	3.8	3.96	3.8	3.93		3.94
Man H5	3.89	8.7	3.84	8.2	3.87	8.3/3.3	3.83	8.3/3.4	3.82
Man H6S	4.25		4.18		4.21		4.18		4.18
Man H6R	3.73		3.75		3.77		–		–
Trp HE3 (H4)	7.65		7.57		7.67		7.65		7.53
Trp HZ3 (H5)	7.13		7.12		7.14		7.14		7.11
Trp HH2 (H6)	7.22		7.21		7.20		7.20		7.20
Trp HZ2 (H7)	7.44		7.44		7.41		7.42		7.42

See also Tables S2 and S7.

a Digested peptides from human RNase 2.

b Values match the ones previously reported by Hofsteenge et al. (1994).

c The 316β–322β chymotryptic peptide from human interleukin-12. Values taken from Doucey et al. (1999).

d The 24–38 tryptic peptide (MSPW^Man^SEWSQCDPCLR) from C9 of human complement system. Values taken from Hofsteenge et al. (1999).

e J_3’4’_+J_4’5’_.

Rotating-frame Overhauser Effect (ROE) analysis of the RNase peptides reveals no signals between H1 and H4 of the mannosyl residue, which excludes all the conformations presenting a short distance between H1 and H4 (^1,4^B, ^1^S_5_, and ^1^S_3_) (see Table S3). The strong ROE signal between H1 and H6 allows elimination of conformations with a long distance between these two protons, i.e., B_2,5_ and ^0^S_2_, and indicates that the hydroxymethyl group is not in its preferred equatorial position_._

The best agreement with the experimental ROE data is obtained for the ^1^C_4_ chair conformation, which has a predicted *J*_3,4_ value of 2.8 Hz. However, this is significantly different from the experimental *J*_3,4_ value of 5.3 Hz (see Table S3); therefore the data presented here are not in agreement with a single conformation of the mannose. Consequently, the C-linked mannopyranosyl residue most likely exists in an ensemble of conformations, among which ^1^C_4_ seems to be the most populated. It could be argued that the normally preferred ^4^C_1_ conformation may not be stable owing to the preference of the rigid Trp ring to adopt an equatorial position that minimizes steric interactions and forces the hydroxymethyl group out of its preferred equatorial position (De Beer et al., 1995). It should be noted that the J-couplings of the mannosyl residue do not match one single conformation; therefore, the mannose ring is supposed to maintain some dynamics in all these peptides. ROE spectra of RNase 2 peptides show no signals between the *C-*mannopyranosyl residue and the neighboring amino acid side chains, and no evidence of long-range connectivities between amino acids or secondary structure, arguing against a significant stabilization of the analyzed peptides. These findings agree with data previously reported for the MSPW^Man^SEWSQCDPCLR fragment, which has no defined structure and presents *J* couplings typical of random coils (Hofsteenge et al., 1999). In conclusion, all the investigated peptides are presumably disordered, and their primary structure does not influence the conformation of the (*C*^*2*^-α-D-Man-)Trp moiety.

### NMR Studies of *C-*Mannose in Native and Denatured RNase 2

Previously, NMR measurements on RNase 2 showed that the mannose residue adopts different orientations around its C-linkage in the native and denatured protein (Löffler et al., 1996). In the native protein, Man H1 is closer to Trp HE1 (Löffler et al., 1996). However, in the denatured protein rotation around the Trp-Man bond brings Man H1 close to Trp HB1/HB2 and Trp HA, analogously to what was detected for the peptides isolated from RNase 2 (Löffler et al., 1996; De Beer et al., 1995) (see Table 2). The three-dimensional structure of native RNase 2 seems to induce a specific orientation of the mannose residue.

**Table 2:** ROEs/NOEs Observed *C-*Mannopyranosyl Moiety in Native and Denatured RNase 2

	Native	Denatured/Isolated Peptides
Man H3–Man H5		aWeak, reported by De Beer et al. (1995)
Man H1–Man H6		aStrong, reported by De Beer et al., (1995) bReported by Löffler et al. (1996)
Man H4–Man H6		aMedium, reported by De Beer et al. (1995)
Man H2–Man H3		aStrong, reported by De Beer et al. (1995)
Man H1–Trp HE1	Reported by Löffler et al. (1996)	
Man H1–Trp HA		aReported by De Beer et al. (1995) bReported by Löffler et al. (1996)
Man H1–Trp HB1/HB2		aReported by Hofsteenge et al. (1994) bReported by Löffler et al. (1996)
Man H2–Trp HE1		aReported by Hofsteenge et al. (1994)
Man H2–Trp HB1/HB2	Reported by Löffler et al. (1996)	bReported by Löffler et al. (1996)
Man H6–Trp HE1	Reported by Löffler et al. (1996)	
Man H2-Val128(Me)	Reported by Vliegenthart and Casset (1998)	

See also Table S3.

a Isolated peptide.

b Denatured Protein.

Analysis of TOCSY (Total Correlation Spectroscopy) and NOESY (Nuclear Overhauser Effect Spectroscopy) experiments of native and denatured RNase allowed assignment of ^1^H chemical shifts for the *C*-mannopyranosyl residue (see Table 3). Values for the denatured protein are close to the ones reported for the digested peptides (see Table 1), whereas the native protein differs significantly for the chemical shifts of H2 and H6S. It can therefore be concluded that the three-dimensional structure of the protein affects the conformational features of the *C*-mannopyranosyl residue, probably through long-range connectivities. In fact, NOE signals identified interactions between Man H2-Val128(Me) and between Man H3 and HB2 of an Asp residue, which could not be undoubtedly attributed to either Asp112 or Asp115 due to overlap of NMR signals that prevented full protein assignment.

**Table 3:** ^1^H Chemical Shifts (ppm) at 300 K of the *C*-Mannopyranosyl Moiety in Native and Denatured RNase 2

	Native	Denatured
Man H1	5.27	5.23
Man H2	4.26	4.45
Man H3	4.13	4.14
Man H4	3.91	3.99
Man H5	ND	3.98
Man H6S	4.55	4.27
Man H6R	3.74	3.76

### Conformational Analysis of Model Compounds and Glycopeptides

In order to gain a detailed insight into the conformational preferences of the *C*^2^-α-D-mannopyranosyl-L-tryptophan moiety (compare Figure 2) a variety of model systems with increasing complexity were studied. First, we used *C*^2^-α-D-mannopyranosyl-3-methyl-indole as a basic model compound to investigate the correlation between ring shape and preferred orientation of the *C-*glycosidic torsion ϕ_H_ (H1-C1-CD-NE1). Subsequently, a conformational analysis of (*C*^*2*^-α-D-Man-)Trp and FTW^Man^AQW was performed.

**Figure 3:**
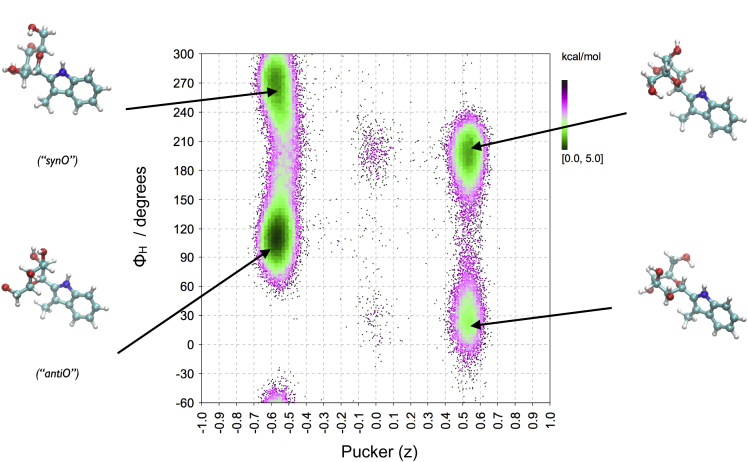
Conformational Preferences of *C*^2^-α-D-mannopyranosyl-3-methyl-indole Glycosidic torsion ϕ_H_ as a function of ring pucker coordinate z. Values from 2 μs gas phase MD simulation using TINKER/MM3 (ε = 4) at 400 K. See also Figures S1–S4

An efficient method to study ring flexibility of carbohydrates is high-temperature MD simulation using TINKER/MM3 with a dielectric constant of four. This method gives direct access to free energy profiles or conformational maps based on the Boltzmann equation (Frank et al., 2007). Plotting the z-component of the ring pucker versus the glycosidic torsion ϕ (Figure 3) clearly shows that there is a dependency between the two parameters: if the mannose adopts a ^4^C_1_ conformation torsion ϕ_H_ prefers values around 110° (termed “synC” here, because the Man-C2 atom is approximately in a syn-orientation to the N-atom of the indole moiety) and 270° (“antiC”), whereas for the ^1^C_4_ conformation the energy minima are located at about 20° (“synH”) and 190° (“antiH”). Consequently, a ring inversion is accompanied by a change of the preferred orientation of the glycosidic torsion. A free energy analysis of the trajectory using the Boltzmann equation reveals that the ring inversion barriers are in the range of 7–8 kcal/mol and that the ^4^C_1_ ring conformation is preferred by about 2.0 kcal/mol (Figure S1). Consequently, according to MM3 force field calculations, the bulky 3-methyl-indole substituent can be well accommodated in an axial orientation at the anomeric C*-*atom. In order to investigate further the effect of substitution at C1 on ring conformation, additional mannosides were also simulated (Figure S1), and it was found that the energy difference between the ^1^C_4_ and ^4^C_1_ conformation follows as expected the order β-D-Man-OMe > α-D-Man-OMe > α-D-Man-3-methyl-indole > α-D-Man-Trp. However, even for *C*^2^-α-D-mannopyranosyl-L-tryptophan the energy for ^1^C_4_ is still higher than for ^4^C_1_. Since this appeared to be not in agreement with results from NMR, we investigated the source of the disagreement in more detail. A series of QM energy calculations at the DFT(b3lyp) and LMP2 levels confirmed that in the gas phase the ^4^C_1_ conformation is preferred over ^1^C_4_ for *C*^2^-α-D-mannopyranosyl-3-methyl-indole (Table S4). However, when the energies were calculated with the PBF solvation model ^1^C_4_ becomes preferred over ^4^C_1_. This means that, in addition to the ring substitution pattern, the solvent may have a profound effect on the conformational equilibrium. To confirm this finding, we investigated conformational energies also based on MD simulations in explicit solvent. Since MM3 (as implemented in TINKER) is not parameterized for use in explicit solvent we used AMBER (which includes GLYCAM06 parameters for the mannose) and GAFF as implemented in YASARA. Also, with these force fields ^4^C_1_ is preferred over ^1^C_4_ when the calculations are performed in the gas phase (Figure S2). However, when MD simulations are performed with the AMBER force field in explicit solvent ^1^C_4_ is preferred by about 1 kcal/mol (Figure S3), independent of the simulation temperature used. The pucker(z) profiles derived at 370, 400, and 500 K are very similar. However, although sampling accumulated 62 μs at 310 K the population of the “skew/boat” conformational states is probably not converged as can be seen from the energy difference of about 1.5 kcal/mol. In general, the ring transition barriers are 1–2 kcal/mol lower than those derived from gas phase simulations. The lowest energy conformational state for *C*^2^-α-D-mannopyranosyl-3-methyl-indole is predicted to be ^1^C_4_-antiH (ϕ_H_ ≈ 200°) (Figure S4).

**Figure 4:**
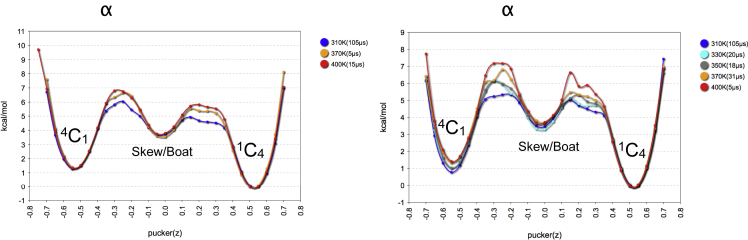
Conformational Ring Transition Energy Profiles of (*C*^*2*^-α-D-Man-)Trp Calculated in explicit solvent at various temperatures (AMBER, NPT/NVT ensemble). See also Figure S5

Sampling of accumulated 105 μs at 310 K for (*C*^*2*^-α-D-Man-)Trp and the glycopeptide FTW^Man^AQW seems to be sufficient to get converged ring pucker energy profiles. The differences to the profiles obtained at higher temperature are limited to the transition state regions (Figure 4). Finally, the conformational analysis of the glycopeptide FTW^Man^AQW revealed that the results are very similar to those obtained for (*C*^*2*^-α-D-Man-)Trp. This means that the peptide does not influence significantly the conformational preferences of the glycosylation site. The lowest energy conformational state for the (*C*^*2*^-α-D-Man-)Trp moiety is predicted to be ^1^C_4_-antiH (ϕ_H_ ≈ 200°) (Figure S5), which is in excellent agreement with NMR results.

**Figure 5:**
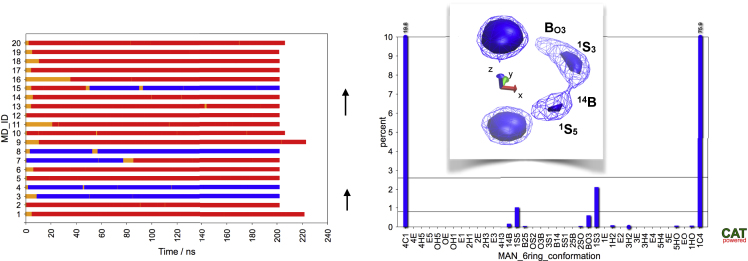
Stability Check of “Skew/Boat” Conformations for Glycopeptide FTW^Man^AQW Based on MD simulations in explicit solvent at 310 K (20 × 0.2 μs, AMBER, NPT ensemble). Left: Simplified representation of ring conformation trajectories (orange: skew/boat, red: ^1^C_4_, blue: ^4^C_1_). Right: population of ring conformational states. Insert: Iso-contour plot indicating the location of the minima in ring pucker space.

Although most of the NMR results would be in agreement with a ^1^C_4_ ring conformation, the ^3^J coupling constant for Man H3/H4 is clearly an indication that other conformations need to be present as well. Therefore, in order to estimate the stability of skew/boat conformations, 20 MD simulations were performed where the starting ring conformation was ^1^S_5_ (Figure 5). In all simulations a transition to a chair conformation occurred in less than 40 ns. Those simulations that were started in ^1^S_5_-synH typically underwent a transition to ^1^C_4_; however, for ^1^S_5_-antiH also a significant number of transitions to ^4^C_1_ occurred. This behavior can also be seen in the pucker(z) versus ϕ_H_ plot shown in Figure S5: skew/boat conformations with ϕ_H_ ≈ 200° (antiH) seem to have a low energy path to either ^1^C_4_ or ^4^C_1_, whereas skew/boat conformations with ϕ_H_ ≈ 10° (synH) seem to have only a low energy transition path leading to ^1^C_4_. In general, the most frequently occurring skew/boat conformation is ^1^S_3_. As previously outlined, the experimentally determined ^3^J_H3/H4_ of 5.3 Hz cannot be explained by a ^1^C_4_ conformation alone (predicted ^3^J_H3/H4_ = 2.8 Hz, compare Table S2). The existence of a significant amount of ^4^C_1_ (predicted ^3^J_H3/H4_ = 8.0 Hz) and/or ^1^S_3_ (predicted ^3^J_H3/H4_ = 8.9 Hz) conformations in the conformational ensemble could in principle explain an increased average ^3^J_H3/H4_ value. However, based on the energy values only about 10% ^4^C_1_ and <1% skew/boat conformations would be predicted to be present in the conformational ensemble, which would not be sufficient to raise the average value of ^3^J_H3/H4_ from 2.8 to 5.3 Hz. Additionally, taking into account a significant amount of ^4^C_1_ or ^1^S_3_ would also influence the average values of the other ^3^J constants, and for many of them the agreement with the experimental values would get worse. Therefore, it is difficult to explain the origin of the increased ^3^J_H3/H4_ of 5.3 Hz satisfactorily based on the results from the conformational analysis of the glycopeptide.

### Conformational Analysis of RNase 2

**Figure 6:**
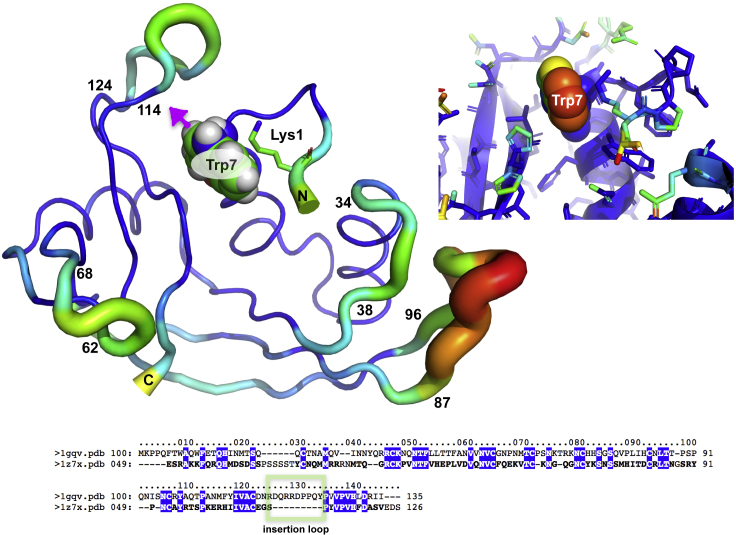
Crystal Structure of Eosinophil-Derived Neurotoxin (EDN, RNase 2) Based on pdb code 1gqv (Swaminathan et al., 2002). Top left: Protein flexibility (beta factor) is indicated by the thickness and color of the backbone representation (PyMol “putty” representation). Top right: Crystallographic beta factors mapped to the atoms as a color code. Red color indicates a high beta factor value. TRP7 is shown as CPK. The arrow denotes the attachment point of α-mannose (*C*-glycosylation site). Bottom: Structure-based sequence alignment (Jung and Lee, 2000) of RNase 1 (sequence taken from PDB entry 1z7x) and RNase 2 (sequence taken from PDB entry 1gqv).

Several crystal structures of Eosinophil-Derived Neurotoxin (EDN, alternative name is RNase 2) are available in the Protein Data Bank (PDB). The entry with the highest resolution was used as a basis for modeling (pdb entry 1gqv, resolution 0.98 Å) (Swaminathan et al., 2002). RNase 2 contains four cysteine bridges stabilizing the protein fold as well as five surface-exposed N-glycosylation sites (Asn 17, 59, 65, 84, and 92). The *C-*glycosylation site (Trp7) is located at the start of the first α helix (residues 7–16). The orientation of the Trp7 indole ring is stabilized by hydrophobic interactions with Lys1. However, analysis of the beta factors indicates significant flexibility of the Trp7 side chain as well as the existence of several flexible loops (Figure 6). The loop with the highest beta factors (residues 87–96) is involved in binding of the placental ribonuclease inhibitor (Iyer et al., 2005).

**Figure 7:**
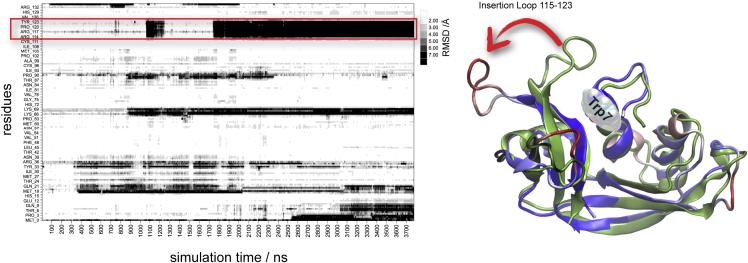
Dynamics of RNase 2 as Shown from MD Simulations on the Microsecond Timescale Left: “RMSD per residue” trajectory plot. Major conformational change (shown by a change to black coloring) of the insertion loop (residue 115–123, indicated by the boxed area) occurs at 1.1 (reversible) and 1.8 μs (irreversible). Right: RNase 2 shown as a cartoon representation. The starting structure of the MD is shown in green. The last snapshot of the trajectory is superimposed and colored by RMSD (blue-white-red, interval 0–7 Å). The change of the insertion loop orientation and the location of Trp7 are indicated. See also Figure S6.

*C-*Mannosylation of Trp7 will most likely have an effect on the dynamics of the Trp7 side chain and the loop consisting of residues 115–123 (termed the “insertion loop” because these residues are not present in RNase 1) and/or the N terminus (residues 1–6) (Figure 6). Since beta factors give only an indication of protein dynamics in the crystal environment, the dynamics of RNase2 in solution was investigated by MD simulation in explicit solvent on the microsecond timescale. In total 23 μs were sampled, and in general the observed dynamics agrees well with the beta factor values of the crystal structure; however, in some of the MD simulations performed significant, but mostly reversible, conformational changes occur in the insertion loop (Figure S6). As an example, the “RMSD per residue” plot of one of the MD simulations (3.7 μs) is shown in Figure 7. It can be observed that the first conformational change, occurring after about 1 μs , was reversible (with a lifetime about 200 ns). However, when the conformational change occurred the second time (at about 1.8 μs) it was not reversible for at least 2 μs. Finally, significant conformational changes occurred also in the N terminus, which made it unlikely that the RNase would recover the crystallographic conformation within a feasible simulation time, so this simulation was not continued. That major conformational changes can occur in the insertion loop was additionally confirmed with MD simulations using other force fields such as OPLS-AA and CHARMM (data not shown); by consequence this result is not an artifact of the Amber force field used.

**Figure 8:**
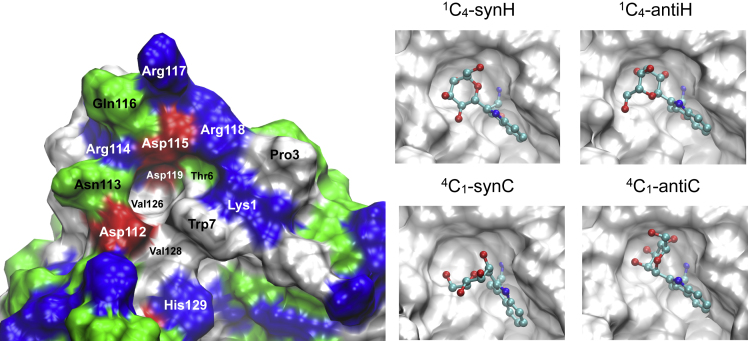
Surface Representation Showing the Cleft Formed between Trp7 and the “Insertion Loop” (Asp112–Arg118) Left: Amino acids are colored by type: negatively charged (red), positively charged (blue), polar (green), lipophilic (white). Right: α-Mannose attached to Trp7 in various conformational states (see text for details). Atoms are colored by element: carbon (cyan), oxygen (red), nitrogen (blue).

**Figure 9:**
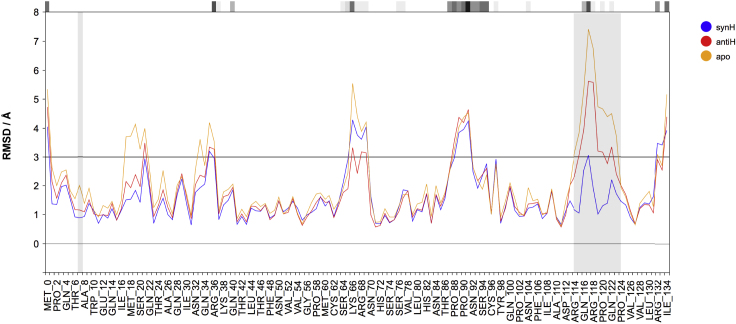
Differences in Average RMSD (C-Alpha Atoms) between ^1^C_4_-Mannosylated RNase and “Apo” RNase 2 A slight reduction in RMSD occurs for Trp7 (highlighted with gray background), but a significant decrease in RMSD is evident for the insertion loop (residues 114–124, highlighted with gray background) when α-mannose is linked in a ^1^C_4_-synH conformation. The X-ray beta factors of the residues are indicated as a grayscale annotation bar above the plot (darker gray means higher value). See also Figure S7.

In the next step an α-mannose residue was linked to Trp7 and it was examined whether the presence of the α-mannose would stabilize the insertion loop and also the N-terminal residues. In general, it was found that the α-mannose moiety could be accommodated without steric clashes and in all four conformational states (^1^C_4_-synH, ^1^C_4_-antiH, ^4^C_1_-synC, ^4^C_1_-antiC) in the cleft formed between Trp7 and Asp112–Arg118 (Figure 8). Therefore, multiple MD simulations were started for each of these conformational states in order to investigate their stability and dynamics. MD simulations revealed that a significant stabilization of the protein occurred only when the α-mannose was linked in a ^1^C_4_-synH conformation. The insertion loop maintained its conformation significantly better than in the simulations without α-mannose (apo) or with α-mannose in a ^1^C_4_-antiH conformation (Figure 9).

When the simulations are performed with mannose in a ^1^C_4_-antiH conformation the mannose residue and the insertion loop are significantly more dynamic. During the 12 μs sampled several reversible transitions to skew/boat conformations occurred. Such transitions did not occur during the 10 μs sampled with mannose in a ^1^C_4_-synH conformation (Figure S7). In general, it was found that ^4^C_1_ ring conformations of C-linked mannose are significantly less stable in RNase 2 than in the glycopeptide FTW^Man^AQW. An extensive analysis involving more than hundred MD simulations of *C-*mannosylated RNase 2 revealed that ^4^C_1_-synC is more stable than ^4^C_1_-antiC, and typically irreversible transitions to ^1^C_4_ occurred always in less than 100 ns (Figures S8 and S9). In contrast, for the glycopeptide the chair form ^4^C_1_ was found to be stable sometimes longer than 1 μs. An additional extensive investigation consisting of 60 MD simulations on the stability of skew/boat forms revealed that ^1^S_3_ is the most populated skew/boat form and that the lifetime of skew/boat forms is four times longer when ϕ_H_ is in syn orientation (Figure S10). In general, the MD data reveal that in the context of the RNase 2, the lifetime of ^4^C_1_ is significantly shorter than in the free glycopeptide and the skew/boat conformational state does have a similar lifetime.

**Figure 10:**
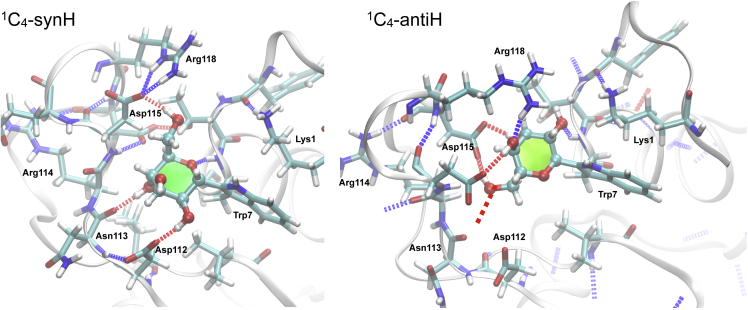
C-Linked α-mannose (^1^C_4_) Forms an Extensive Network of Hydrogen Bonds with RNase 2 See also Tables S5 and S6.

It should be noted that during the accumulated 22 μs sampled for αMan(^1^C_4_)-RNase 2 no transition between antiH and synH occurred. Since also the ^4^C_1_/^1^C_4_ transitions were irreversible on the microsecond timescale this means that it is currently unfeasible to directly determine the free energy landscape of the various conformational states of *C-*mannosylated RNase 2 by population statistics using the Boltzmann equation. However, the extensive MD data clearly show that ^1^C_4_-synH is the most stable conformational state of *C-*mannosylated RNase 2: within the accumulated 10 μs sampled for αMan(^1^C_4_-synH)-RNase2 the α-mannose remained in this state and formed several very stable hydrogen bonds with the protein (Figure 10, Tables S5, and S6). Of particular interest are the hydrogen bonds between Man O6 and Asp115/Arg118, which directly stabilize the conformation of the insertion loop. The CH-π interactions between the indole moiety of Trp7 and the aliphatic side chain CH atoms of Lys1 are likely to be critical stabilization factors for the conformation of the N-terminal residue. Since the H-bond between the ring oxygen and Trp7 backbone nitrogen stabilizes directly the side chain conformation of Trp7, the existence of the C*-*linked α-mannose has most likely also an indirect stabilization effect on the N terminus (residues 1–6) of RNase 2.

Finally, the conformational state ^1^C_4_-synH in native RNase is also in excellent agreement with NMR results (Tables 1 and S7).

## Discussion

The function of *C-*mannosylation has not been fully elucidated, but studies conducted so far indicate that it plays an important role in protein folding, targeting of substrate proteins, or regulating cellular signaling (Ihara et al., 2015). Hartmann and Hofsteenge (2000) supposed that the 14 mannose residues linked to properdin could be exposed at the surface of the protein thanks to their hydrophilic character and therefore mediate properdin interaction with the multivalent mannose-binding lectin present in serum. Differently from properdin, in RNase 2 the accessibility of the mannose residue is quite poor. In RNase 2 and EDN*,* the presence of *C-*mannose seems not to be related directly to any specific biological function. Comparison of enzymatic activity of recombinant RNase from *Escherichia coli* with the *C*-mannosylated RNase 2 could not elucidate significant differences (Furmanek and Hofsteenge, 2000). Similarly, *C*-mannose does not seem to be essential for the neurotoxic activity of EDN, since the related RNase eosinophil cationic protein, which contains an arginine at position 7 instead of (*C*^*2*^-α-D-Man-)Trp, is equally toxic (Snyder and Gleich, 1997).

Biophysical and biochemical studies show that glycosylation facilitates the folding and increases the stability of some proteins (Kassenbrock et al., 1988; Imperiali and Rickert, 1995), and *C*-mannosylation of thrombospondin type 1 repeats imparts higher resistance to thermal and reductive denaturation processes (Shcherbakova et al., 2019). Therefore, it could be hypothesized that C-linked mannose may similarly exert a structural function in RNase 2.

(*C*^*2*^-α-D-Man-)Trp is located in the N-terminal α-helix of RNase 2. The three dimensional structure of rEDN has been compared with that of bovine pancreatic RNase A, which lacks the (*C*^*2*^-α-D-Man-)Trp moiety (Mosimann et al., 1996). Although the α helices and the β strands are almost identical in the two proteins, the N-terminal loop and the large insertion loop are absent in RNase A. NMR and molecular simulation studies demonstrated that the presence of *C-*mannose stabilizes the conformation of the large insertion loop and also the N-terminal loop. In principle, the *C-*mannose can be accommodated in the cleft formed between Trp 7 and Asp 112–118 without steric clashes and links the N-terminal residues with residues 112–115 through a specific network of hydrogen bonds in a “lock-and-key”-like manner. In this study we investigated the conformational preferences of *C*-mannosylated tryptophan and its impact on the stability of RNase 2 by NMR and extensive MD simulation. In NOESY spectra, an intense NOE between mannose H1 and H6 clearly indicates that the hydroxymethyl group is not in the usual equatorial orientation, which is typical of the ^4^C_1_ conformation, but in the axial orientation typical of the ^1^C_4_ chair. Surprisingly, the correct energy ranking of the chair forms could only be obtained when solvent was taken into account in the calculations. Consequently, the bulky axial substituent at the anomeric C*-*atom seems to be insufficient to change the conformational equilibrium to favor ^1^C_4_. In general, the conformational analysis based on MD simulations in explicit solvent has been computationally very challenging (many months of calculation time) because hundreds of microseconds had to be sampled to obtain sufficiently converged results. This has become feasible only recently owing to a significant advance in computer hardware (fast GPUs), MD software (e.g., YASARA) and high-performance analysis software (Conformational Analysis Tools) that allow one to calculate and analyze hundreds of GBytes of trajectory data and assemble the results of hundreds of MD simulations efficiently. It is encouraging that such high-level MD simulations (physiological conditions, sampling on the microsecond timescale) can give valuable insights into the dynamics of glycoproteins that seem to be sufficiently reliable and that can currently not be obtained with any other experimental method.

To achieve an optimal conformational fit, the mannose has to adopt a ^1^C_4_-synH conformational state. NMR and MD simulations show that ^1^C_4_-antiH is preferred in unfolded RNase 2 (and in glycopeptides), which raises the intriguing question how such a conformational change may occur. Microsecond MD simulations show that the insertion loop and the mannose orientation are more flexible for ^1^C_4_-antiH; therefore, the nascently folded RNase 2 may undergo a self-optimization process where the ^1^C_4_-synH and the insertion loop get locked after the antiH/synH conformational transition has occurred.

It may be concluded that the main role of the mannose is to stabilize the N-terminal loop of the protein by moderating the interaction with the large insertion loop, whose presence has been hypothesized to contribute to the antiviral properties of EDN (Domachowske et al., 1998). This hypothesis finds support in the behavior of other glycoproteins, such as the proteinase inhibitor PMP-C, where the fucose residue linked to Thr9 causes a decrease in the number of dynamic fluctuations of the molecule (Mer et al., 1996).

### Limitations of the Study

This study does not provide experimental evidence that *C*-mannosylation enhances the structural stability of RNase 2. Although the MD simulations presented here are very extensive, it is currently technically unfeasible to sample the conformational equilibrium of the different chair conformations of mannose when attached to RNase 2.

### Resource Availability

#### Lead Contact

Johannes F.G. Vliegenthart, j.f.g.vliegenthart@uu.nl.

#### Materials Availability

This study did not generate new unique reagents.

#### Data and Code Availability

The datasets supporting the current study have not been deposited in a public repository because they are too large (terabytes) but are available from the corresponding author on request.

The software Conformational Analysis Tools (CAT) used for analysis of the MD trajectories is developed by Martin Frank and is freely available at http://www.md-simulations.de/CAT/

## Methods

All methods can be found in the accompanying Transparent Methods supplemental file.

## Supplemental Information

Document S1. Transparent Methods, Figures S1–S10, and Tables S1–S7

## Footnotes

Supplemental Information can be found online at https://doi.org/10.1016/j.isci.2020.101371.
