# Supplementary material for: C-Mannosylation Enhances the Structural Stability of Human RNase 2
Source: iScience. 2020 Jul 16;23(8):101371. doi: 10.1016/j.isci.2020.101371 (PMC7399192; doi:10.1016/j.isci.2020.101371)
Supplement: Document S1. Transparent Methods, Figures S1–S10, and Tables S1–S7 [file mmc1.pdf]

iScience, Volume *23*

Supplemental Information

C-Mannosylation Enhances the Structural Stability of Human RNase 2

Martin Frank, Daniela Beccati, Bas R. Leeflang, and Johannes F.G. Vliegenthart

Supplemental Figures

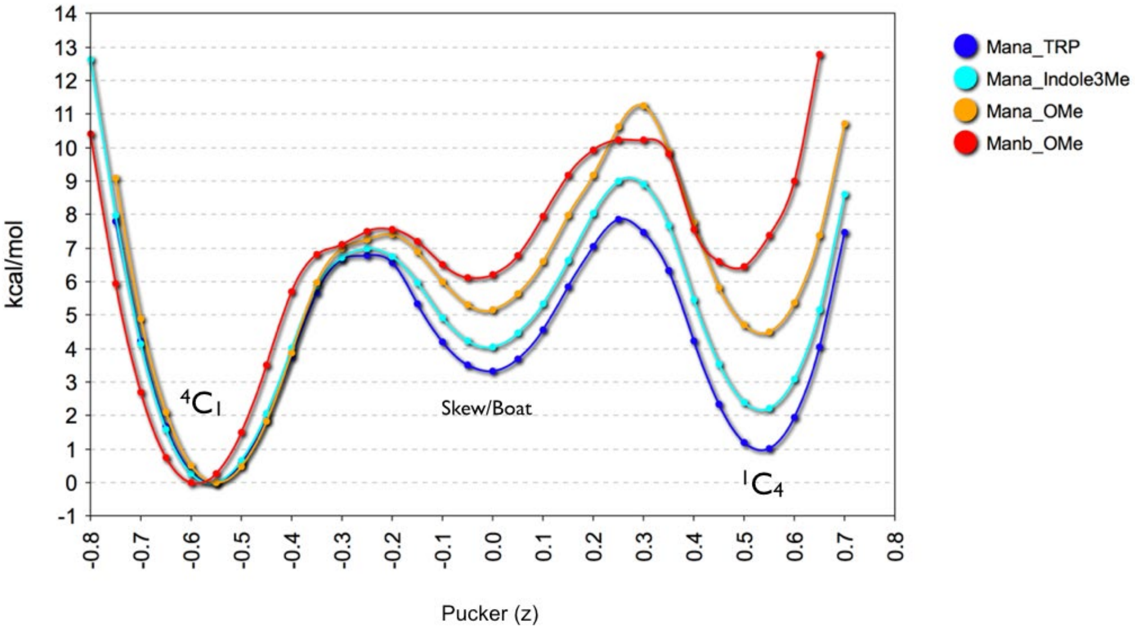

Figure S1: Ring conformational energies as a function of ring pucker coordinate z for various D-mannopyranosyl compounds. Related to Figure 3.

Calculated from gas phase MD simulations using TINKER/MM3 (ε = 4) at 500 K. Energy difference between the $^1C_4$ and $^4C_1$ conformation follows as expected the order β-D-Man-OMe > α-D-Man-OMe > α-D-Man-3-methyl-indole > α-D-Man-Trp.

AMBER14/YASARA 310K

group	counts	E_sys_MD	E_sys_MIN
4C1_synC	200	5.0 ± 0.9	1.5 ± 0.7
4C1_synO	200	0.0 ± 1.0	0.0 ± 0.1
1C4_antiH	285	6.0 ± 1.6	1.8 ± 0.5
1C4_synH	115	8.7 ± 1.2	4.2 ± 0.2

GAFF/YASARA 310K

group	counts	E_sys_MD	E_sys_MIN
4C1_synC	198	2.1 ± 1.3	0.4 ± 0.6
4C1_synO	202	0.0 ± 0.7	0.0 ± 0.2
1C4_antiH	200	3.3 ± 1.5	2.8 ± 0.3
1C4_synH	200	10.3 ± 1.2	7.5 ± 0.1

minimized

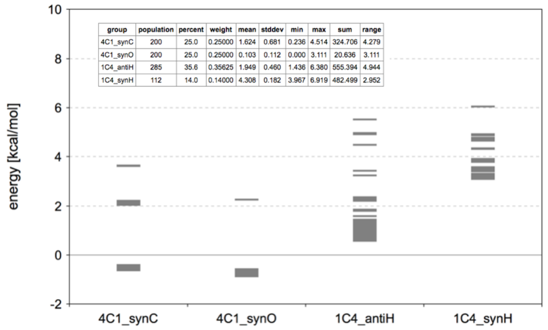

minimized

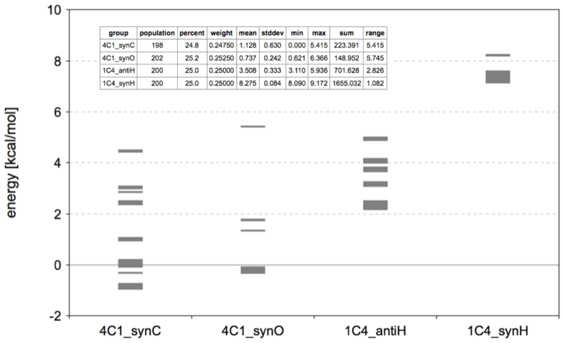

Figure S2: Conformational state energies of *C*^{2}-α-D-mannopyranosyl-3-methyl-indole calculated in the gas phase with AMBER(Glycam-06) and GAFF. Related to Figure 3.

Values are derived by calculating the relative force field energies of the MD snapshots directly and after energy minimization. The values are grouped into the four conformational states shown in Figure 3. $^4C_1$(synO) has consistently the lowest average force field energy. However, for GAFF some of the minimized frames belonging to the $^4C_1$(synC) state have lower energy than the energy minimum of the $^4C_1$(synO) states. It is obvious that there are significant differences in the minimized energies within a conformational state, which is most likely caused by different orientations of the OH-groups of the mannose. This highlights the difficulties involved in deriving reliable relative force field energies. Despite this it can be concluded that in the gas phase $^4C_1$ is preferred over $^1C_4$ for both force fields used.

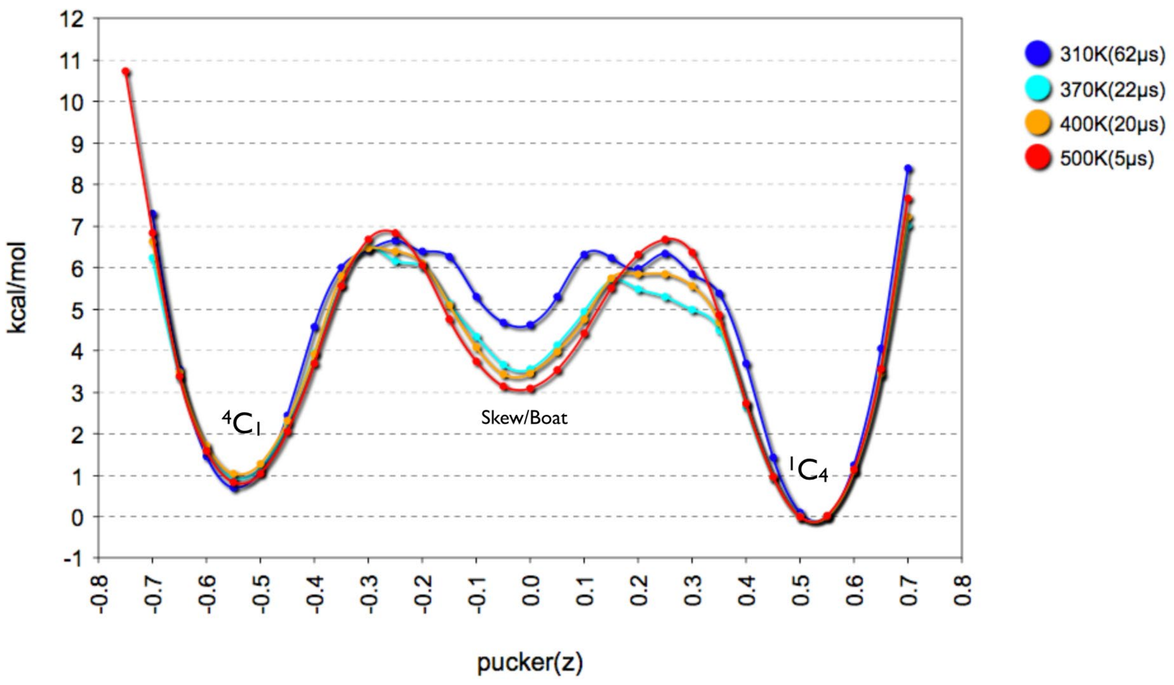

Figure S3: Conformational ring transition profiles of C^2-α-D-mannopyranosyl-3-methyl-indole. Related to Figure 3.
Calculated in explicit solvent at various temperatures (AMBER, NVT ensemble).

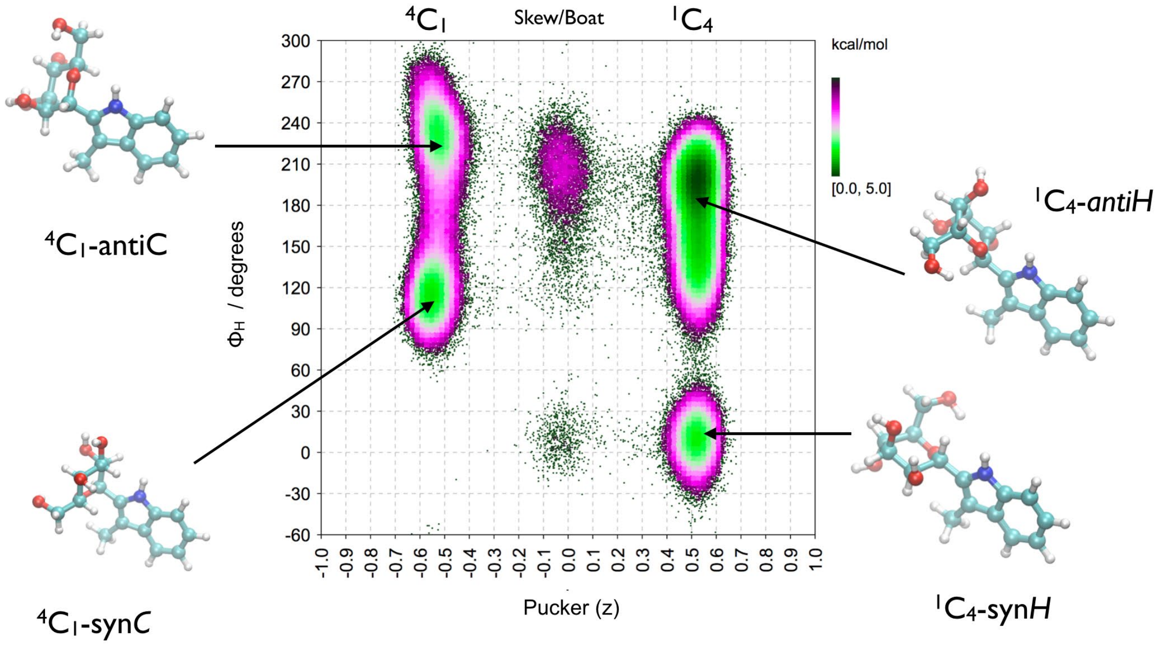

Figure S4: Conformational preferences of C^2-α-D-mannopyranosyl-3-methyl-indole as a function of ring puckering coordinate z and glycosidic torsion φ_H. Related to Figure 3.
Values from 63 μs MD simulation in explicit solvent at 310 K (AMBER, NPT ensemble).

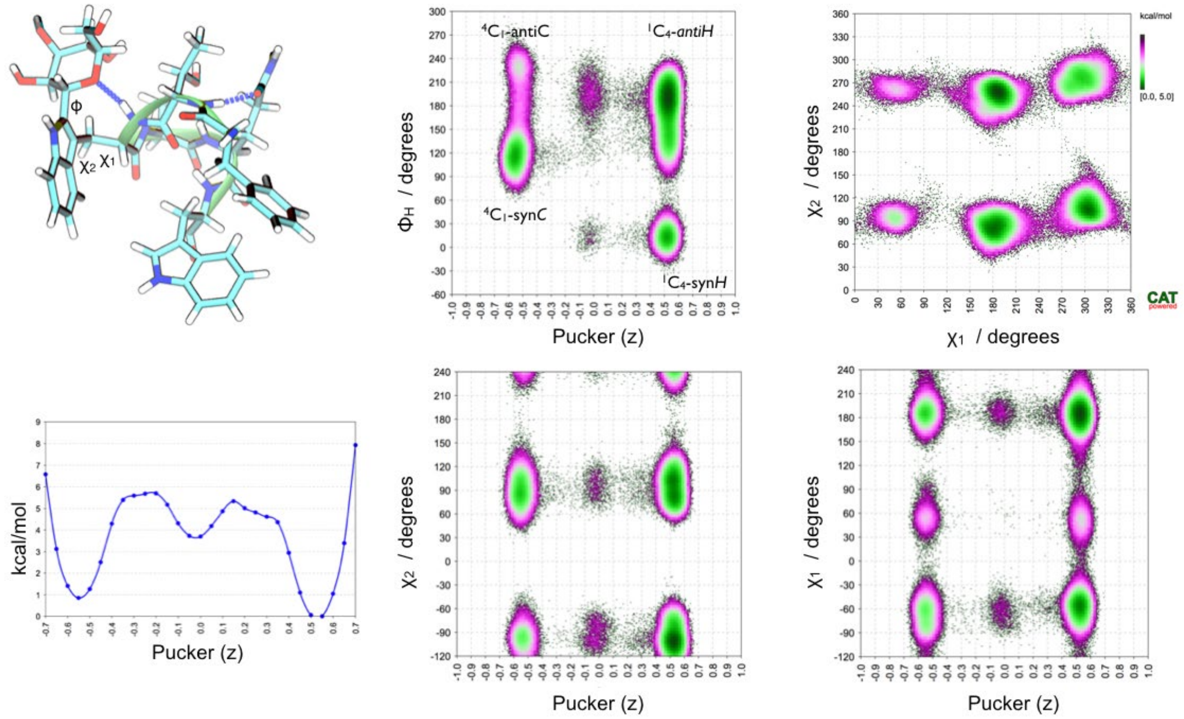

Figure S5: Conformational analysis of FTW^{Man}AQW. Related to Figure 4.
Calculated from MD simulation in explicit solvent at 310 K (105 µs, AMBER, NPT ensemble). χ_1 and χ_2 do not seem to be significantly inclueneced by the ring conformation of mannose.

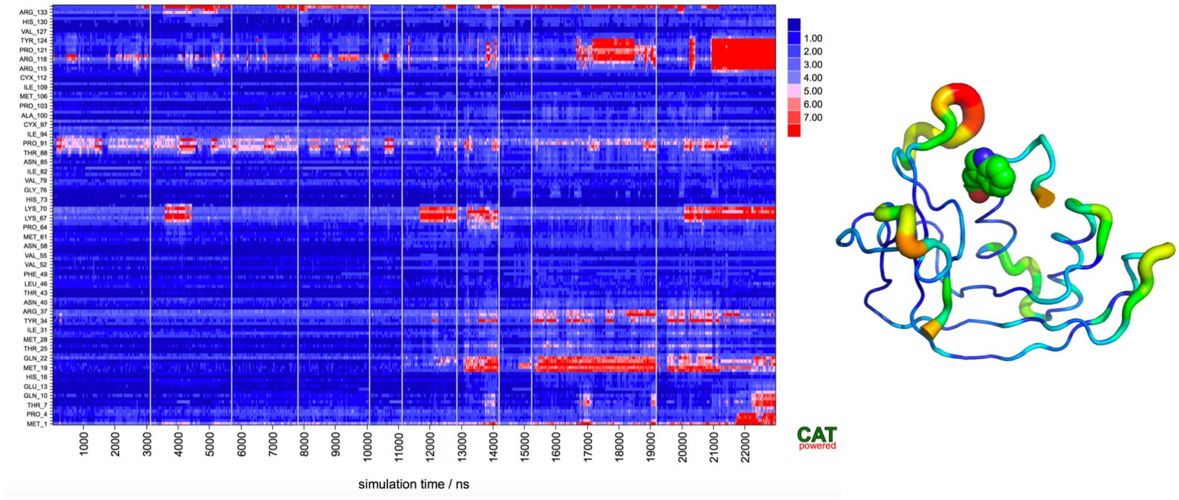

Figure S6: Stability check of RNase 2. Related to Figure 7.
Based on MD simulations in explicit solvent at 310 K (23 µs, AMBER, NPT ensemble). Left: accumulated RMSD per residue plot (individual trajectories are separated by a vertical line). Right: average residue RMSD as PyMol ‘putty’ representation.

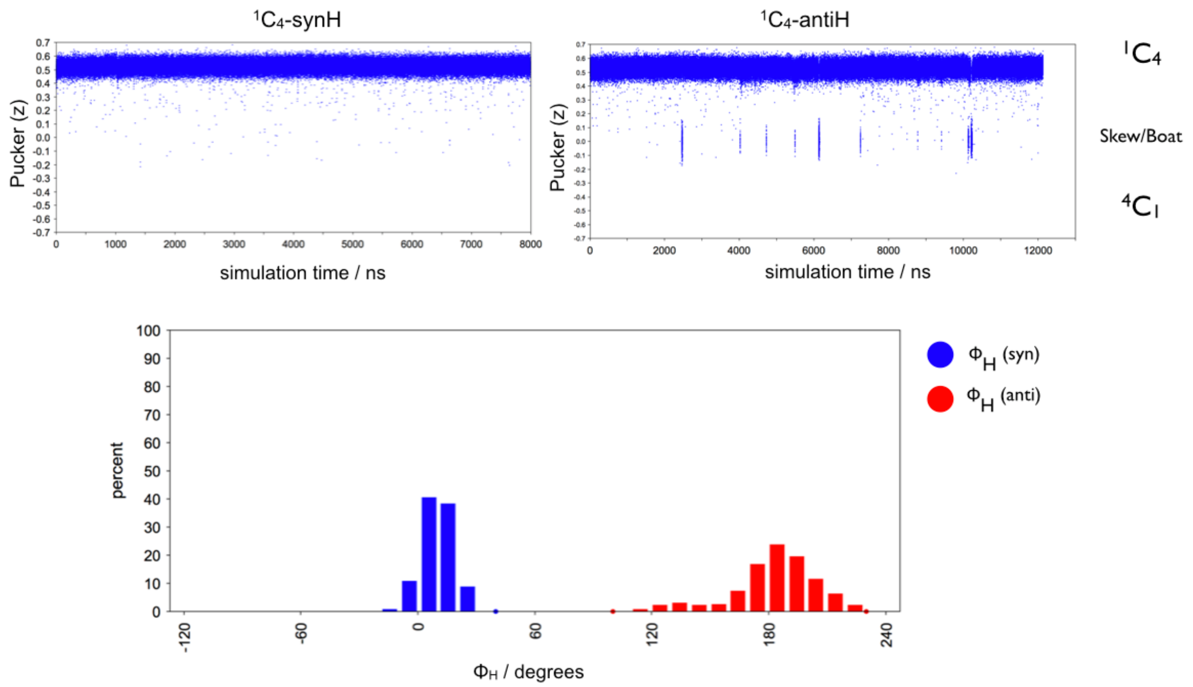

Figure S7: Stability check of ring conformations in αMan($^1C_4$)-RNase 2. Related to Figure 9.
Based on MD simulations in explicit solvent at 310 K (22 µs, AMBER, NPT ensemble). MD simulations are started with mannose in $^1C_4$-synH and $^1C_4$-antiH, respectively. It should be noted that φ_H in state antiH is more flexible and only during the simulation started from state $^1C_4$-antiH transitions to 'skew/boat' occurred.

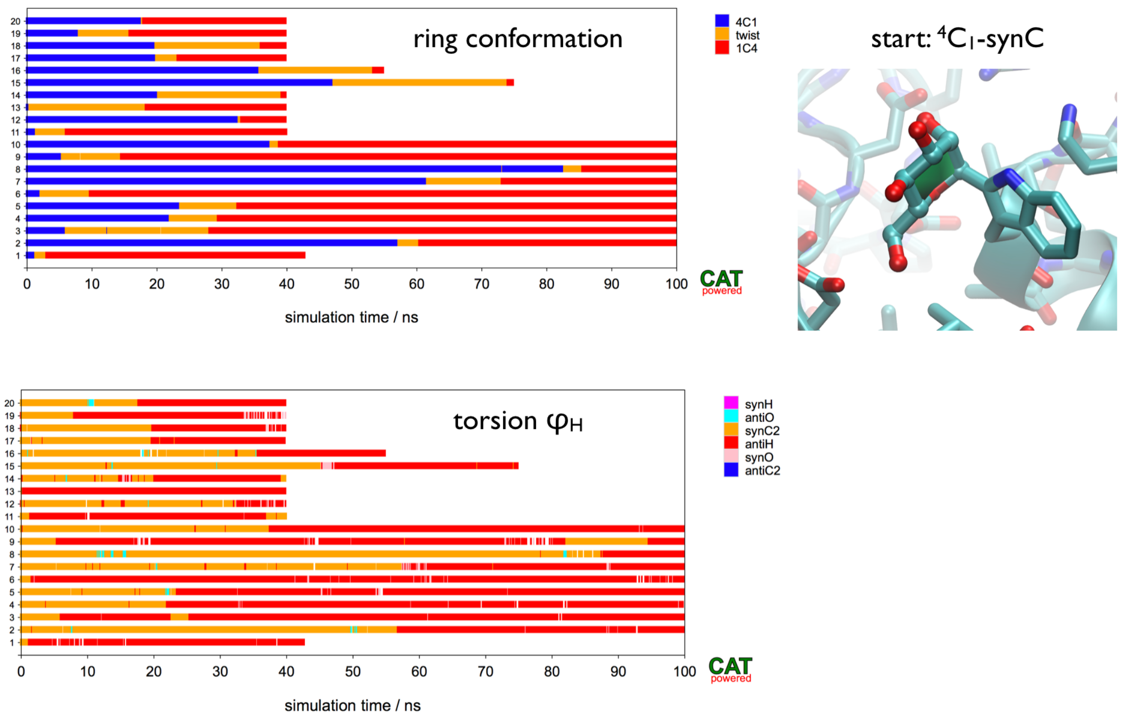

Figure S8: Stability check of αMan($^4C_1$-synC)-RNase 2.
Based on 20 MD simulations in explicit solvent at 310 K (AMBER, NPT ensemble). In all simulations a conformational transition to $^1C_4$ occurred in less than 100 ns. It should be noted that the change of the chair form is accompanied by a change of the glycosidic torsion φ_H.

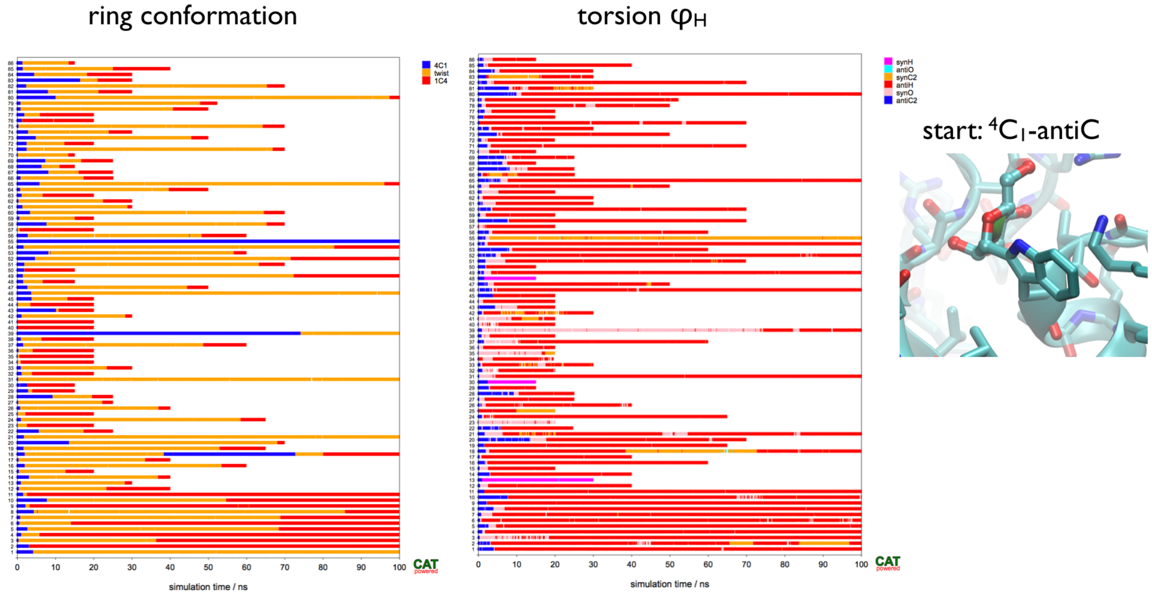

Figure S9: Stability check of αMan($^4C_1$-antiC)-RNase 2.
Based on 86 MD simulations in explicit solvent at 310 K (AMBER, NPT ensemble). It should be noted that in simulation 55 the ring form $^4C_1$ is stable for longer than 100 ns, but in this simulation φ_H has changed to synC after about 3 ns.

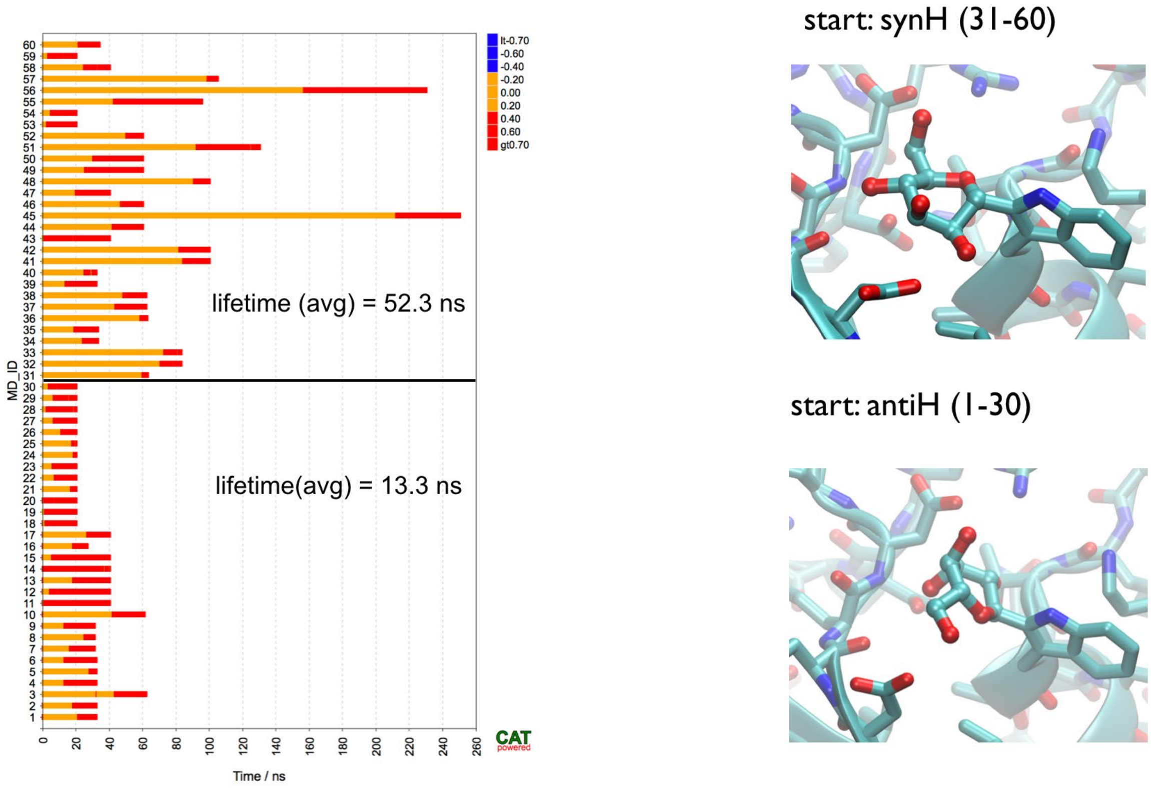

Figure S10: Stability check of αMan('skew/boat')-RNase 2.
Based on 60 MD simulations in explicit solvent at 310 K (AMBER, NPT ensemble). 'Skew/boat' have also a relatively short lifetime, however they are more stable when torsion φ_H is syn.

Supplemental Tables

Table S1: **X-rays structures of C-mannosylated proteins available in the Protein Data Bank (PDB)** (accessed 10/2019). Related to Figure 1.

**Name**	**PDB entries**
MAG	5lfr (2.1Å), 5lfv (2.3Å), 5lfu (4.3Å), 5lf5 (3.8Å)
Interleukin-2 Receptor	5m5e (2.3Å)
Interleukin-21 Receptor	4nzd (2.7Å), 3tgx (2.8Å)
Human Complement Component C6	3t5o (2.9Å)
Human Complement Component C8	3ojy (2.5Å)
Human Complement Component C9	6cxo (2.2Å)
C5b6	4a5w (3.5Å), 4e0s (4.2Å)
Micronemal Protein MIC2	4okr (2.6Å), 4oku (3.2Å)
ADAMTS13	3vn4 (2.8Å)
Properdin	6s08 (2.0Å), 6s0b (2.3Å), 6s0a (2.5Å), 6rus (2.8Å), 6sej (3.5Å), 6rv6 (3.5Å), 6rur (6Å), 6ruv (6.15Å)

Table S2: Experimental and calculated $J_{i,i+1}$ coupling constants.
Comparison between experimental $J_{i,i+1}$ coupling constants for the pyranose ring of Manα1-Trp from Human RNase 2 and $J_{i,i+1}$ coupling constants calculated for selected ring conformations using the generalized Karplus equation (Haasnoot et al. 1980). The following electronegativity factors were used: 1.3 for oxygen, 0.4 for carbon, 0.85 for nitrogen. Related to Table 1.

	**$J_{i,i+1}$ (Hz)**			
	**H1/H2**	**H2/H3**	**H3/H4**	**H4/H5**
experimental	8.2	3.2	5.3	3.4
$^{4}C_{1}$	1.4	3.2	8.0	8.5
$^{1}C_{4}$	7.7	3.1	2.8	1.2
$^{0,3}B$	3.5	3.2	2.5	3.1
$B_{0,3}$	2.8	2.9	8.1	3.7
$^{1,4}B$	7.9	6.7	8.6	8.5
$B_{1,4}$	1.3	6.7	2.2	1.1
$^{2,5}B$	1.4	3.7	1.4	1.2
$B_{2,5}$	7.7	3.1	1.8	8.5
$^{1}S_{5}$	7.7	5.4	6.0	8.3
$^{0}S_{2}$	6.7	2.1	0.8	7.2
$^{3}S_{1}$	2.3	4.4	3.4	1.6
$^{5}S_{1}$	2.0	5.2	0.9	1.5
$^{2}S_{0}$	1.4	2.2	6.6	1.9
$^{1}S_{3}$	5.4	3.8	8.9	7.2

Table S3: **Comparison between experimental ROEs contacts' intensities for the pyranose ring of Manα1-Trp from Human RNase 2 and proton distances calculated for selected ring conformations.**
Related to Table 2.

	Distances (Å)				
	**H3-H5**	**H1-H6**	**H4-H6**	**H2-H3**	**H1-H4**
Experimental	(ROE)W[a]	(ROE)S	(ROE)M	(ROE)S	-
$^{4}C_{1}$	2.6	4.4	2.5	2.4	4
$^{1}C_{4}$	4.4	2.4	2.6	2.4	4.0
$^{0,3}B$	3.5	4.5	2.4	2.3	4.6
$B_{0,3}$	3.2	2.5	2.5	2.4	3.4
$^{1,4}B$	2.3	3.1	2.9	2.2	1.9
$B_{1,4}$	4.2	4.1	2.5	2.2	4.8
$^{2,5}B$	4.1	2.9	2.4	2.9	4.7
$B_{2,5}$	3.3	4.5	2.5	2.4	3.2
$^{1}S_{5}$	2.7	3.8	2.7	2.3	2.3
$^{0}S_{2}$	3.7	4.6	2.4	2.4	4.1
$^{3}S_{1}$	4.2	4.4	2.4	2.3	4.7
$^{5}S_{1}$	4.2	4.1	2.7	2.3	4.8
$^{2}S_{0}$	3.5	2.6	2.4	2.5	4.0
$^{1}S_{3}$	2.7	2.3	2.4	2.3	2.4

[a] ROE intensities were estimated from cross-peak volumes in 2D-ROESY spectra recorded with a mixing time of 100 ms (De Beer et al. 1995). ROE intensities were determined as a percentage of the summed ROE and diagonal-peak intensities in a ω_2-column of the appropriate line width and were classified as weak (w, less than 5%), medium (m, 6-10%), or strong (s, more than 10%).

Table S4: Conformational preferences of C^2-α-D-mannopyranosyl-3-methyl-indole studied by quantum mechanics calculations.
GP_Energy = gas phase energy; dGP_Energy = relative gas phase energy; SP_energy = solution phase energy; dSP_energy = relative solution phase energy; Solv_energy = Solvation Energy; T-Rx: ring torsions, T_NCCO: torsion Man:O5-Man:C1-Trp:CD1-Trp:NE1. Energies are given in kcal/mol.

ID	ring_conf	Task	QM_Method	QM_Basis	GP_Energy	dGP_energy	SP_Energy	dSP_energy	Solv_Energy	T_R1	T_R2	T_R3	T_R4	T_R5	T_R6	T_NCCO
**Mana_IndolMe_1C4synH-B3LYP631ssppPBF**	1C4	Optimization	DFT(b3lyp)/SOLV	6-31g**++	-636253.187	5.500	-636281.187	-2.125	-27.973	46	-40	43	-55	61	-55	146.1
**Mana_IndolMe_1C4synO-B3LYP631ssppPBF**	1C4	Optimization	DFT(b3lyp)/SOLV	6-31g**++	-636256.000	2.687	-636283.187	-4.125	-27.184	47	-41	44	-56	61	-55	-31.5
**Mana_IndolMe_1C4synO2-B3LYP631ssppPBF**	1C4	Optimization	DFT(b3lyp)/SOLV	6-31g**++	-636254.000	4.687	-636279.687	-0.625	-25.623	43	-41	49	-62	62	-51	216.3
**Mana_IndolMe_1C4synO_gg-B3LYP631ssppPBF**	1C4	Optimization	DFT(b3lyp)/SOLV	6-31g**++	-636255.187	3.500	-636282.000	-2.937	-26.811	48	-43	44	-54	57	-52	-22.0
**Mana_IndolMe_1S3synH-B3LYP631ssppPBF**	1S3	Optimization	DFT(b3lyp)/SOLV	6-31g**++	-636256.437	2.250	-636278.250	0.812	-21.788	-27	67	-36	-31	74	-37	94.0
**Mana_IndolMe_1S3synO-B3LYP631ssppPBF**	1S3	Optimization	DFT(b3lyp)/SOLV	6-31g**++	-636257.750	0.937	-636278.812	0.250	-21.063	-23	66	-38	-29	75	-42	-86.5
**Mana_IndolMe_1S3synO2-B3LYP631ssppPBF**	1S3	Optimization	DFT(b3lyp)/SOLV	6-31g**++	-636256.437	2.250	-636279.312	-0.250	-22.885	-30	69	-36	-32	72	-35	94.2
**Mana_IndolMe_4C1synC-B3LYP631ssppPBF**	4C1	Optimization	DFT(b3lyp)/SOLV	6-31g**++	-636257.375	1.312	-636279.062	0.000	-21.702	-55	53	-48	48	-49	50	172.7
**Mana_IndolMe_4C1synO-B3LYP631ssppPBF**	4C1	Optimization	DFT(b3lyp)/SOLV	6-31g**++	-636258.625	0.062	-636279.812	-0.750	-21.160	-54	51	-48	53	-56	54	12.2
**Mana_IndolMe_4C1synO_gt-B3LYP631ssppPBF**	4C1	Optimization	DFT(b3lyp)/SOLV	6-31g**++	-636258.687	0.000	-636279.062	0.000	-20.363	-57	47	-39	46	-56	59	188.0
**Mana_IndolMe_1C4synH-LMP2631ssppPBF_SP**	1C4	"Single Point"	LMP2	6-31g**++	-634347.250	5.437	-634377.125	0.250	-29.918	51	-46	45	-53	59	-56	127.8
**Mana_IndolMe_1C4synO-LMP2631ssppPBF_SP**	1C4	"Single Point"	LMP2	6-31g**++	-634349.937	2.750	-634378.375	-1.000	-28.410	52	-46	45	-53	59	-56	-42.5
**Mana_IndolMe_1C4synO2-LMP2631ssppPBF_SP**	1C4	"Single Point"	LMP2	6-31g**++	-634347.437	5.250	-634374.000	3.375	-26.587	49	-45	46	-55	59	-54	187.5
**Mana_IndolMe_1C4synO_gg-LMP2631ssppPBF_SP**	1C4	"Single Point"	LMP2	6-31g**++	-634348.250	4.437	-634376.250	1.125	-27.983	50	-45	45	-55	59	-55	-41.8
**Mana_IndolMe_1S3synH-LMP2631ssppPBF_SP**	1S3	"Single Point"	LMP2	6-31g**++	-634340.937	11.750	-634372.375	5.000	-31.390	-27	67	-36	-31	74	-37	94.0
**Mana_IndolMe_1S3synO-LMP2631ssppPBF_SP**	1S3	"Single Point"	LMP2	6-31g**++	-634351.187	1.500	-634375.375	2.000	-24.212	-40	64	-25	-38	63	-20	-36.8
**Mana_IndolMe_1S3synO2-LMP2631ssppPBF2_SP**	1S3	"Single Point"	LMP2	6-31g**++	-634343.500	9.187	-634373.500	3.875	-29.986	-25	66	-38	-29	73	-40	115.2
**Mana_IndolMe_1S3synO2-LMP2631ssppPBF_SP**	1S3	"Single Point"	LMP2	6-31g**++	-634349.625	3.062	-634372.500	4.875	-22.872	-32	63	-28	-36	68	-30	181.1
**Mana_IndolMe_4C1synC-LMP2631ssppPBF_SP**	4C1	"Single Point"	LMP2	6-31g**++	-634351.375	1.312	-634376.375	1.000	-25.035	-49	52	-55	59	-56	49	238.0
**Mana_IndolMe_4C1synO-LMP2631ssppPBF_SP**	4C1	"Single Point"	LMP2	6-31g**++	-634352.687	0.000	-634377.375	0.000	-24.690	-52	53	-53	56	-55	51	33.9

Table S5: Hydrogen bonds between α-mannose and RNase 2 for αMan($^{1}C_{4}$-synH). Only those with a frequency > 1% are shown. Related to Figure 10.

Index	Donor	Acceptor	Population	Distance	Angle
1	aDManp:O4	ASN_113:O	99.9	2.7	161
2	TRP_7:N	aDManp:O5	90.8	3.0	151
3	aDManp:O2	ASP_112:OD1	50.4	2.7	166
4	aDManp:O2	ASP_112:OD2	46.3	2.7	166
5	aDManp:O6	ASP_115:OD2	35.9	2.7	163
6	aDManp:O6	ASP_115:OD1	32.9	2.7	163
7	ARG_118:NH2	aDManp:O6	23.1	3.0	140

Table S6: **Hydrogen bonds between α-mannose and RNase 2 for αMan($^{1}C_{4}$-antiH)**. Only those with a frequency > 1% are shown. Related to Figure 10.

Index	Donor	Acceptor	Population	Distance	Angle
1	TRP_7:N	aDManp:O2	55.9	3.0	150
2	aDManp:O6	ASN_113:O	46.5	2.8	154
3	aDManp:O3	ASP_115:OD1	25.0	2.7	164
4	aDManp:O3	ASP_115:OD2	17.3	2.7	164
5	aDManp:O6	ASP_112:OD1	13.9	2.7	162
6	aDManp:O6	ASP_112:OD2	12.3	2.7	162
7	aDManp:O4	ASP_112:OD1	9.0	2.7	165
8	aDManp:O3	ASP_119:OD1	8.8	2.7	163
9	aDManp:O4	ASP_112:OD2	6.8	2.7	165
10	aDManp:O6	ASP_119:OD2	5.7	2.7	166
11	aDManp:O3	ASN_113:O	5.6	2.7	158
12	aDManp:O4	ASP_115:OD2	4.4	2.7	160
13	LYS_1:NZ	aDManp:O2	3.8	3.0	133
14	aDManp:O3	ASP_119:OD2	2.7	2.9	143
15	aDManp:O4	ASP_115:OD1	1.6	2.7	162
16	ARG_118:NH2	aDManp:O3	1.3	3.0	141
17	aDManp:O6	ASP_112:O	1.1	2.9	158

Table S7: Statistics of H-H distances.
Based on MD simulations at 310 K (1 µs, AMBER, NPT ensemble) for C-glycosylated RNase 2 with αMan in $^{1}C_4$ ring conformation. Values are given in Å in the format "mean(stddev)[min,max]". Related to Table 1.

Distance Label	1C4(antiH)	1C4(synH)
MAN(H1-H2)	3.1(0.0)[2.9,3.2]	3.0(0.0)[2.9,3.2]
MAN(H1-H3)	3.8(0.1)[3.4,4.3]	3.8(0.1)[3.3,4.2]
MAN(H1-H4)	4.0(0.1)[3.2,4.6]	4.0(0.1)[3.4,4.5]
MAN(H1-H5)	3.8(0.1)[3.4,4.1]	3.8(0.1)[3.4,4.1]
MAN(H1-H61)	3.2(0.8)[1.7,4.7]	3.7(0.2)[1.7,4.5]
MAN(H1-H62)	2.5(0.5)[1.6,4.6]	2.2(0.2)[1.7,4.2]
MAN(H3-H5)	4.3(0.1)[3.9,4.6]	4.3(0.1)[4.0,4.5]
MAN(H4-H5)	2.6(0.1)[2.1,2.9]	2.6(0.1)[2.2,2.9]
MAN(H4-H61)	2.7(0.3)[1.9,4.0]	2.4(0.2)[1.9,3.9]
MAN(H4-H62)	3.3(0.4)[2.0,4.2]	3.1(0.2)[2.0,4.1]
MAN(H1)-TRP7(HB1)	2.1(0.1)[1.7,3.1]	3.9(0.1)[3.6,4.4]
MAN(H1)-TRP7(HB2)	3.6(0.1)[2.8,4.5]	5.1(0.1)[4.7,5.4]
MAN(H1)-TRP7(HA)	4.4(0.3)[3.3,5.4]	5.4(0.1)[4.8,6.1]
MAN(H1)-TRP7(HE1)	3.8(0.1)[3.3,4.1]	2.5(0.1)[2.0,3.0]
MAN(H2)-TRP7(HB1)	4.7(0.2)[3.5,5.4]	2.3(0.2)[1.8,3.1]
MAN(H2)-TRP7(HB2)	5.9(0.2)[4.9,6.3]	3.5(0.2)[2.6,4.3]
MAN(H2)-TRP7(HA)	5.8(0.2)[4.6,7.0]	5.1(0.2)[4.5,5.8]
MAN(H2)-TRP7(HE1)	2.6(0.2)[1.8,3.7]	4.4(0.1)[3.5,4.9]
MAN(H61)-TRP7(HE1)	6.0(0.4)[4.6,7.1]	6.0(0.3)[3.7,6.9]
MAN(H62)-TRP7(HE1)	5.5(0.2)[4.4,6.7]	4.4(0.3)[3.3,6.6]
MAN(H2)-VAL128(CG1)	7.5(0.6)[5.0,11.2]	3.7(0.6)[2.5,6.7]
MAN(H2)-VAL128(CG2)	8.6(0.6)[5.6,10.9]	4.9(0.6)[2.6,6.8]
MAN(H3)-ASP112(HB1)	9.0(0.8)[4.8,12.5]	4.4(0.4)[2.0,7.1]
MAN(H3)-ASP112(HB2)	8.1(0.9)[4.5,11.6]	3.2(0.4)[1.9,5.5]
MAN(H5)-ASP115(HB1)	7.5(1.4)[2.2,13.6]	2.2(0.2)[1.8,3.5]
MAN(H5)-ASP115(HB2)	7.8(1.0)[3.1,13.6]	3.8(0.2)[2.3,5.3]

Transparent methods

Sample purification. RNase 2 (E.C. 3.1.27.5) was a gift of J. Hofsteenge and purified from male human urine by chromatography on SP-Sephadex at pH 3.0, heparin Sepharose, and SP-Sephadex at pH 7.5. Final purification was achieved by reversed-phase HPLC, using a C4 column (Vydac, Hispania, CA) equilibrated in 0.1% trifluoroacetic acid. Protein was eluted with a linear gradient of 10.5—56% CH_3CN in 80 min, at a flow rate of 1 mL/min (Hofsteenge et al. 1994). Glycopeptides from the N-terminal region of RNase were prepared by digestion of reduced and carboxymethylated protein with the protease from *Staphylococcus aureus* (2% W/W; 18 h), thermolysin (3% W/W; 18 h), elastase (5% W/W; 4 h), or aminopeptidase M (10 units/nmol of peptide; 2h). All digestions were performed in 50 mM NH_4HCO_3, pH 7.8, at 37°C. Subsequently, glycopeptides were purified by reverse phase HPLC on a Vydac C_{18} column (4.6 X 250 mm) employing eluent A: 0.1% trifluoroacetic acid; eluent B: 0.085% trifluoroacetic acid, 70% acetonitrile. The flow rate was 1.0 ml/ min. The column was washed isocratically for 5 min with 3.5% acetonitrile after which the gradient (0.15% acetonitrile/min) was started (Hofsteenge et al. 1991).

NMR Spectroscopy. NMR spectra were recorded with a Bruker AMX-500 or AMX-600 spectrometer (Bijvoet Center, Department of NMR Spectroscopy, Utrecht University, The Netherlands), and a Varian UnityPlus 750 MHz spectrometer (SON NMR Large Scale Facility, Utrecht University). Native RNase 2 was dissolved in 90% (v/v) H_2O/D_2O, 0.1M NaCl, 1mM NaN_3, pH 5.1. NOESY and TOCSY ^{1}H-NMR spectra were recorded at 300, 315 and 320 K. 2D NOESY spectra(Jeener et al. 1979) were recorded with a mixing time of 100 ms, with 350 increments (in the t1 dimension) of 2K data points, with 96 scans per increment. Water resonance was suppressed by irradiating at low power during the relaxation delay and the mixing time. In addition, a spoil sine-bell-shaped pulsed field gradient (PFG) was applied. 2D TOCSY spectra (Griesinger et al. 1988) were recorded with a clean MLEV-17 spin lock sequence with a duration of 10, 50, or 80 ms. Spectra were recorded with 440 increments (in the t1 dimension) of 2K data points each, with 64 scans. As for NOESY experiments, water resonance was suppressed by a low power irradiation applied during the relaxation delay, and a spoil sine-bell-shaped pulsed field gradient (PFG) was included. Temperature was lowered by 3 K to avoid changes of chemical shifts due to the heat released when the sequence is applied. For all 2D experiments, quadrature detection was achieved using the State-TPPI method (Marion et al. 1989). NMR data sets were processed using NMRPipe (Delaglio et al. 1995) and TRITON NMR software packages (Bijvoet Center, University of Utrecht). REGINE software package (Bijvoet Center, University of Utrecht) was used to analyse NMR data and to perform protein assignments.

Peptides were dissolved in 5mM potassium phosphate, pH 5.4 in D_2O (99.96 atom% D, Isotec, USA). Spectra were recorded at a probe temperature of 300 K. Chemical shifts are expressed in ppm relative to internal acetone (^{1}H δ 2.225; ^{13}C δ 32.910). Suppression of the water resonance was achieved by a water eliminated Fourier Transform pulse (WEFT-pulse) sequence (Hard et al. 1992). For 1D ^{1}H-NMR spectra 16-512 free induction decays of 8K or 16K complex data points were collected. 2D spectra were acquired with 256-512 experiments of 2048 data points, with 32-240 scans per increment. Quadrature detection in the t_1 dimension was achieved by either the time-proportional phase increment (TPPI) method (Marion & Wüthrich 1983), or the States-TPPI method. 2D TOCSY experiments were recorded using a MLEV 17 mixing sequence of 20, 50 or 60 ms (Braunschweiler & Ernst 1983; Bax & Davis 1985) preceded by a trim pulse of 2.5 ms. Typically, TOCSY spectra were recorded with a mixing time of 10, 50, or 100 ms with the spin-lock field strength adjusted for a 90° pulse-length of 29-30 μs. 2D ROESY spectra were obtained with a spin-lock pulse length of 100 ms, 300 ms, 750 ms, or 1.2 s. The spin-lock field strength was in accordance with a 90° pulse of 100-120 μs. The frequency offset was initially placed on the HOD resonance and switched to about 5.7 ppm just before application of the spin-lock pulse, thereby reducing the Hartmann-Hahn transfer during the ROESY mixing time (Leeflang & Kroon-Batenburg 1992). NMR data sets were processed using ProspectND software (Bijvoet Center, University of Utrecht). Briefly, the final matrix size was zero-filled to 2Kx1K or 4Kx2K and multiplied with phase-shifted (squared-)sine-bell function prior Fourier transformation.

Molecular Modeling. The starting structures for simulations of human RNase 2 and glycopeptides were prepared based on PDB entry 1gqv (resolution 0.98 Å) using the graphical interface of YASARA (Krieger & Vriend 2014). The protonation state of amino acids was adjusted to pH 7.4 (Krieger et al. 2012). Simulations in explicit solvent (using periodic boundary conditions) were generally performed in 0.1% NaCl solution using YASARA with GPU acceleration (Krieger & Vriend 2015). Simulations at 300 K and 310 K were performed under NPT conditions. For simulations at higher temperatures the system was first equilibrated at 310 K and then the sampling was performed under NVT conditions. HTMD simulations without explicit solvent were performed with TINKER (https://dasher.wustl.edu/tinker/) using MM3 (Allinger et al. 1990). For these simulations a

dielectric constant of four was used to mimic condensed phase conditions. Conformational Analysis Tools (CAT, http://www.md-simulations.de/CAT/) was used for analysis of trajectory data, general data processing, generation of scientific plots in SVG format, and for assignment of MM3 atom types. Torsion angles are defined as φ (Man:O5-Man:C1-Trp7:CD1-Trp7:NE1), φ_H (Man:H1-Man:C1-Trp7:CD1-Trp7:NE1), χ_1 (Trp7:N-CA-CB-CG), χ_2 (Trp7:CA-CB-CG-CD1). Quantum mechanics calculations were performed with Jaguar (Version 8.1, Schrödinger, LLC). Open source Pymol (Version 1.8x, Schrödinger, LLC) and VMD (Humphrey et al. 1996) was used to generate molecular graphics.
In total more than 100 μs were sampled at various temperatures for the solvated RNase system (apo, C-mannosylated) and more than 500 μs for the glycopeptide and the model compounds (see Table below).

Overview on timescales sampled in the project

	RNASE2_apo	RNASE2_1C4	RNASE2_4C1	FTWAQW_Man	Trp_Man	Man_Indole
300 K		60 ns	4.8 μs	10 μs	3.3 μs	
310 K	23 μs	23 μs	28 μs	105 μs	116 μs	63 μs
330 K	1.1 μs	2.5 μs	3 μs	20 μs		25 μs
350K	0.5 μs	0.2 μs	0.5 μs	20 μs	2.5 μs	23 μs
370 K	3.6 μs	18 μs	10.7 μs	31 μs	5 μs	33 μs
400 K	0.3 μs	0.4 μs	0.2 μs	5 μs	17 μs	31 μs
Sum	28 μs	44 μs	47 μs	191 μs	143 μs	175 μs

In general the sampling was distributed over multiple independent simulations that were run in parallel on standard computer equipment, for example: Amber16 (PMEMD.cuda, GeForce GTX 680, RNase 2, 25713 atoms, 2 fs timestep, 66 ns/day), Gromacs 2018.2 (OPLSAA, i7-6900K CPU 3.20GHz / GeForce GTX 980, 26462 atoms, 2 fs timestep, 155 ns/day), YASARA 18.4 (AMBER03, i7-6900K CPU 3.20GHz/GeForce GTX 980, 26256 atoms, 2.5 fs timestep, 160 ns/day), YASARA 18.4 in 'fast mode' (AMBER03, i9-7920X CPU 2.90GHz/ GeForce GTX 1080 Ti, 26256 atoms, 5 fs timestep, 335 ns/day).
As can be estimated based on the example performances given for the various computer systems above, a significant amount of calculation time was consumed in the project and several Terabytes of accumulated trajectory data was output in XTC format. Individual MD simulations were typically run for at least 1 μs with a maximum of 10 μs. The distribution of the sampling over many independent simulations required the development of an efficient analysis workflow using Conformational Analysis Tools that allows to monitoring the conformational changes that are occurring in the individual simulations, and that is also able to accumulate the data of multiple MDs in order to obtain combined statistical results.

Supplemental References

Allinger, N.L., Rahman, M. & Lii, J.H., 1990. A Molecular Mechanics Force-Field (MM3) for Alcohols and Ethers. *Journal of the American Chemical Society*, 112(23), pp.8293–8307. Available at: http://pubs.acs.org/doi/abs/10.1021/ja00179a012.

Bax, A. & Davis, D.G., 1985. Practical aspects of two-dimensional transverse NOE spectroscopy. *Journal of Magnetic Resonance (1969)*, 63(1), pp.207–213.

Braunschweiler, L. & Ernst, R.R., 1983. Coherence transfer by isotropic mixing: Application to proton correlation spectroscopy. *Journal of Magnetic Resonance (1969)*, 53(3), pp.521–528.

Delaglio, F. et al., 1995. NMRPipe: a multidimensional spectral processing system based on UNIX pipes. *Journal of Biomolecular NMR*, 6(3), pp.277–293.

Griesinger, C. et al., 1988. Clean TOCSY for proton spin system identification in macromolecules. *Journal of the American Chemical Society*, 110(23), pp.7870–7872.

Haasnoot, C.A.G., de Leeuw, F.A.A.M. & Altona, C., 1980. The relationship between proton-proton NMR coupling constants and substituent electronegativities—I: An empirical generalization of the karplus equation. *Tetrahedron*, 36(19), pp.2783–2792.

Hard, K. et al., 1992. The Asn-linked carbohydrate chains of human Tamm-Horsfall glycoprotein of one male. Novel sulfated and novel N-acetylgalactosamine-containing N-linked carbohydrate chains. *European Journal of Biochemistry*, 209(3), pp.895–915.

Hofsteenge, J., Servis, C. & Stone, S.R., 1991. Studies on the interaction of ribonuclease inhibitor with pancreatic ribonuclease involving differential labeling of cysteinyl residues. *J Biochem*, 266(35), pp.24198–24204.

Humphrey, W., Dalke, A. & Schulten, K., 1996. VMD: Visual molecular dynamics. *Journal of Molecular Graphics*, 14(1), pp.33–8– 27–8.

Jeener, J. et al., 1979. Investigation of exchange processes by two-dimensional NMR spectroscopy. *The Journal of chemical physics*, 71(11), pp.4546–4553.

Krieger, E. & Vriend, G., 2015. New ways to boost molecular dynamics simulations. *Journal of Computational Chemistry*, 36(13), pp.996–1007.

Krieger, E. & Vriend, G., 2014. YASARA View—molecular graphics for all devices—from smartphones to workstations. *Bioinformatics*.

Krieger, E. et al., 2012. Assignment of protonation states in proteins and ligands: combining pKa prediction with hydrogen bonding network optimization. *Methods Mol Biol*, 819, pp.405–421.

Leeflang, B.R. & Kroon-Batenburg, L.M.J., 1992. CROSREL: Full relaxation matrix analysis for NOESY and ROESY NMR spectroscopy. *Journal of Biomolecular NMR*, 2(5), pp.495–518.

Marion, D. & Wüthrich, K., 1983. Application of phase sensitive two-dimensional correlated spectroscopy (COSY) for measurements of 1H-1H spin-spin coupling constants in proteins. *Biochem. Biophys. Res. Comm.*, 113(3), pp.967–974. Available at: http://ukpmc.ac.uk/abstract/MED/6307308.

Marion, D. et al., 1989. Rapid recording of 2D NMR spectra without phase cycling. Application to the study of hydrogen exchange in proteins. *Journal of Magnetic Resonance (1969)*, 85(2), pp.393–399.
